# Poly-γ-Glutamic Acid Complexed With Alum Induces Cross-Protective Immunity of Pandemic H1N1 Vaccine

**DOI:** 10.3389/fimmu.2019.01604

**Published:** 2019-07-11

**Authors:** Quyen Thi Nguyen, Chaewon Kwak, Wang Sik Lee, Jaemoo Kim, Jinyoung Jeong, Moon Hee Sung, Jihyun Yang, Haryoung Poo

**Affiliations:** ^1^Infectious Disease Research Center, Korea Research Institute of Bioscience and Biotechnology, Daejeon, South Korea; ^2^Department of Biosystems and Bioengineering, KRIBB School of Biotechnology, University of Science and Technology, Daejeon, South Korea; ^3^Environmental Disease Research Center, Korea Research Institute of Bioscience and Biotechnology, Daejeon, South Korea; ^4^Department of Nanobiotechnology, KRIBB School of Biotechnology, University of Science and Technology, Daejeon, South Korea; ^5^Department of Bio and Nanochemistry, Kookmin University, Seoul, South Korea

**Keywords:** vaccine adjuvant, influenza virus, efficacy, cross-protection, antibody-dependent cellular cytotoxicity, cytotoxic T lymphocyte activity

## Abstract

The use of a good vaccine adjuvant may induce a higher immunogenicity profile of vaccine antigens. Here, we developed a new adjuvant by combining poly-γ-glutamic acid (γ-PGA) with alum (PGA/Alum) and investigated its ability to enhance the immunogenicity and the cross-reactive efficacy of pandemic H1N1 (pH1N1) influenza vaccine antigen. PGA/Alum enhanced antigen delivery to draining lymph nodes and antigen-specific immunogenicity in mice using OVA as a model antigen. It also greatly increased OVA-specific antibody production, cytotoxic T lymphocyte (CTL) activity, and antibody-dependent cellular cytotoxicity (ADCC). These abilities of PGA/Alum improved the protective efficacy of pH1N1 vaccine antigen by increasing hemagglutination-inhibition titers, enhancing ADCC and CTL activity, and speeding viral clearance following homologous viral challenge. Importantly, the cross-protective efficacy of pH1N1 vaccine against heterologous viruses [A/Puerto Rico/8/34 (H1N1) and A/Hong Kong/1/1968 (H3N2)] was significantly enhanced by PGA/Alum, and cross-reactive ADCC and CTL activities were observed. Together, our results strongly suggest that PGA/Alum may be a promising vaccine adjuvant for preventing influenza and other infectious diseases.

## Introduction

Traditional vaccines are composed of killed or attenuated viruses or bacteria and have several drawbacks, including safety concerns, the need for complicated culture of the infectious agents, and the low yields of their manufacturing processes (1, 2). To solve these problems, researchers have developed new types of vaccine such as subunit recombinant vaccines and DNA vaccines (3). Unfortunately, most of these vaccines are unable to generate sufficient antigen-specific immunogenicity to effectively prevent infectious diseases. In particular, influenza continues to occur as a seasonal epidemic and in sporadic pandemics with considerable morbidity and mortality worldwide, largely because we lack an effective vaccine capable of inducing broad cross-protection against newly emerging influenza viruses that underwent antigenic drift and shift (4). The future development of effective influenza vaccines has been proposed to hinge on the use of adjuvants that improve the immunogenicity and cross-reactive immunity of vaccine antigens (5, 6).

Despite extensive research, relatively few adjuvants have been licensed for use with human vaccines. These adjuvants included aluminum salts (alum) and emulsions (e.g., MF59) (6, 7). Since the 1920s, alum has been used as a vaccine adjuvant for a wide range of vaccines in the US and Europe. It can trigger robust humoral immune responses (i.e., antibody production), but does little to enhance the antibody-dependent cellular cytotoxicity (ADCC) and cytotoxic T lymphocyte (CTL) activities that are critical for the protection against various pathogens, including viruses and intracellular pathogens, and cross-reactivity against heterologous influenza viruses (8–11). Regarding MF59, its adjuvanted influenza vaccine did not show any significant difference in the cross-reactivity compared to the unadjuvanted vaccine (12), and MF59 itself has been associated with adverse effects, including injection site pain and the induction of inflammatory arthritis (13).

The current approach for developing vaccine adjuvants is based on our knowledge of the innate immune responses that initiate adaptive immune responses. Some adjuvants include agonists that can enhance innate immune responses through pattern recognition receptors, such as toll-like receptors (TLRs) (14, 15). Poly-γ-glutamic acid (γ-PGA) is a natural, biodegradable, and edible biopolymer composed of repeating units of both D- and L-glutamic acids combined via γ-amide linkages that is secreted naturally by the *Bacillus subtilis sups. Chungkookjang* commonly found in Korean traditional soybean paste, chungkookjang (16). We previously reported that γ-PGA induces TLR4-mediated innate immune responses and robustly provokes Th1 immune responses to enhance CTL activity (17). Taking advantage of the safety and potential immunostimulatory properties of γ-PGA, we developed a vaccine adjuvant by combining γ-PGA with alum (PGA/Alum) to resolve the limitations of currently licensed vaccine adjuvants. We investigated the physiochemical properties, efficacy, and action mechanisms of PGA/Alum using the model antigen, ovalbumin (OVA), *in vivo* and *in vitro*. We then evaluated the adjuvant efficacy of PGA/Alum in improving pandemic H1N1 (pH1N1) influenza vaccine antigen-specific cellular immune responses, antibody (Ab) production, and cross-reactivity against heterologous influenza A viruses. Our results demonstrate that PGA/Alum increased dendritic cell (DC) activation and antigen trafficking, thereby enhancing adaptive immune responses, particularly antigen-specific CTL activity and ADCC. Furthermore, the protective and cross-reactive efficacies of pH1N1 influenza vaccine were substantially improved by PGA/Alum, which conferred cross-protection accompanied with cross-reactive ADCC and CTL activities. Together, our results strongly suggest that PGA/Alum may be a promising vaccine adjuvant for prevention of influenza-related and other infectious diseases.

## Materials and Methods

### Mice

Six- to eight-week-old female C57BL/6 mice (Orient Bio) were housed in a specific pathogen-free (SPF) facility in the Korea Research Institute of Bioscience and Biotechnology (KRIBB). Handling of mice and experimental procedures were reviewed and approved by the Institutional Animal Care and Use Committee (IACUC) of the KRIBB (KRIBB-AEC-17013 and KRIBB-AEC-17162) and were performed according to the Guidelines for Animal Experiments of the KRIBB.

### Cells

Bone marrow-derived DCs (BMDCs) were generated and maintained in RPMI 1640 (Gibco) that contained 10% heat-inactivated FBS (Gibco), 100 U/ml penicillin, and 100 mg/ml streptomycin (Gibco), as previously described (18). B16F10 and MDCK cells were purchased from ATCC and maintained in DMEM (Gibco) that contained 10% heat-inactivated FBS, 100 U/ml penicillin, and 100 mg/ml streptomycin. B16mOVA cells (B16F10 cells expressing membrane-bound OVA) were kindly provided by Dr. David J. Mooney (Harvard University, USA) and were maintained in DMEM supplemented with 10% heat-inactivated FBS, 100 U/ml penicillin, 100 mg/ml streptomycin, and 1 μg/ml puromycin dihydrochloride (Millipore).

### Preparation of PGA/Alum

PGA/Alum was prepared by combining γ-PGA (BioLeaders) and Imject alum (Thermo Fisher) in a 0.9% saline solution. Briefly, 1 mg/ml alum solution, pH 6.5 (adjusted by HCl) was added drop-wise into 1 mg/ml γ-PGA solution, pH 6.8 (adjusted by ammonia solution) (v:v = 1:1) with constant stirring at 70 × *g*. The resultant PGA/Alum was collected by centrifugation at 15,000 × *g* for 30 min at 4°C, re-suspended in a 0.9% saline solution, and stored at 4°C prior to use.

### Preparation of Viruses

The influenza viruses A/California/04/09 (pH1N1), A/Puerto Rico/8/34 (H1N1), and A/Hong Kong/1/68 [H3N2 (a reassortant H3N2 virus carrying the HA and NA genes from A/Hong Kong/1/68 and internal genes from A/Puerto Rico/8/34)], were grown in 9 to 10-day-old SPF embryonated chicken eggs (Orient Bio) for 48 h at 37°C. The viruses were harvested from the allantoic fluids by centrifugation at 3,500 × *g* for 10 min at 4°C and filtration through 0.45 μm pore-size membrane filter (Millipore) and then stored at −80°C until use. All viral experiments were performed under biosafety level 2+ (BSL2+) conditions.

### Dynamic Light Scattering (DLS)

The hydrodynamic diameter and polydispersity index of alum and PGA/Alum were measured using a particle-size analyzer (ELS-Z; Otsuka Inc.). Zeta-potential values were measured with a Zeta-sizer (Nano ZS; Malvern Instruments Ltd.).

### Scanning Electron Microscopy (SEM)

The morphologies of alum and PGA/Alum were observed using a field-emission scanning electron microscope (FE-SEM, Quanta 250 FEG). Briefly, alum and PGA/Alum (100 μg/ml, 1 ml) were dispersed in autoclaved saline buffer, dropped and dried on a silicon wafer, coated with gold for 60 s using a Polaron SC7640 sputter coater (Quorum Technologies Ltd.) and then subjected to SEM.

### Transmission Electron Microscopy (TEM)

TEM images of alum and PGA/Alum were obtained using a field-emission transmission electron microscope (FE-TEM; JEOL Ltd.). For visualization, alum and PGA/Alum (100 μg/ml) solutions were dropped and dried on a formvar- and carbon-coated copper grid (Ted Pella, Inc).

### Fourier Transform Infrared (FT-IR) Spectroscopy

To analyze the chemical structure of PGA/Alum, we performed FT-IR analysis using FT-IR spectroscopy (Alpha-T; Bruker Optics). Alum and PGA/Alum solutions (1 mg/ml in saline buffer) were centrifuged at 10,000 × *g* for 5 min and re-dispersed in distilled water. After all solutions were dried under a vacuum for 2 days, the powder was mixed with potassium bromide (KBr). FT-IR signals were obtained by scanning from 500 to 4,000 cm^−1^ with a scan resolution of 4 cm^−1^.

### Determination of OVA Antigen Loading

PGA/Alum and alum (10 mg/ml each) were mixed overnight with OVA antigen (3 mg/ml; Sigma-Aldrich) at 4°C with constant rotation at 15 × *g*. The resulting PGA/Alum-OVA complexes were centrifuged at 14,000 × *g* for 15 min, and the supernatants were collected for the analysis of unloaded OVA. The amount of loaded OVA was calculated based on the amount of unloaded OVA in the supernatants, as assessed via BCA protein assay (Pierce).

### Cell Viability Assay

Immature BMDCs were generated from bone marrow cells of C57BL/6 mice and stimulated with alum, γ-PGA, or PGA/Alum at 25, 50, 100, or 200 μg/ml for 24 h. The cell viability was measured using the Cell Counting Kit-8 (Dojindo Laboratories) according to the manufacturer's instructions.

### *In vitro* Activation and Antigen Uptake/Processing of BMDCs

To investigate BMDC activation, we stimulated the cells with 100 μg/ml alum, 100 μg/ml γ-PGA, or 200 μg/ml PGA/Alum for 24 h at 37°C. To examine the antigen uptake and processing of BMDCs, we incubated the cells with either 5 μg/ml FITC-OVA (Thermo Fisher) or 5 μg/ml DQ-OVA (a self-quenching conjugate of OVA that exhibits bright green fluorescence upon proteolytic degradation; Thermo Fisher) mixed with or without alum, γ-PGA, or PGA/Alum for 1 or 5 h at 37°C, respectively.

### ELISA

The cytokine levels in the culture supernatants were measured using ELISA kits (BD Bioscience) according to the manufacturer's instructions. To determine the levels of antigen-specific IgG Ab in the sera of immunized mice, ELISA plates (Nunc) were coated overnight with 1 μg/ml OVA protein or 0.5 μg/ml influenza vaccine antigen (A/California/7/2009 NYMC X-179A H1N1; provided by Mogam Biotechnology Research Institute) in carbonate solution, pH 9.5, at 4°C. ELISA was performed as previously described (18, 19).

### Flow Cytometry

All cells were blocked with anti-CD16/CD32 monoclonal Ab (mAb) and stained with the subsequently described fluorochrome-conjugated mAbs. The mAbs were purchased from BD Biosciences, BioLegend, or eBioscience. To measure DC activation, cells were stained with PE-conjugated mAbs against mouse CD40, CD80, CD86, MHC class II molecules or isotype-matched control mAbs. To examine the proportions of DCs located at the injection sites and draining lymph nodes (dLNs), C57BL/6 mice were intramuscularly (i.m.) immunized with 5 μg Alexa Fluor 647-conjugated OVA mixed with or without 800 μg PGA/Alum, and cells were obtained after 3, 6, 12, and 48 h post-immunization. In other experiments, C57BL/6 mice were i.m. immunized with 5 μg Alexa Fluor 647-conjugated OVA alone or mixed with 400 μg alum, 400 μg γ-PGA, or 800 μg PGA/Alum, and cells were obtained after 24 h post-immunization. The cells were stained with APC eFluor 780-conjugated anti-CD11c and Alexa Fluor 488-conjugated anti-MHC class II mAbs. To measure frequency of OVA_257−264_ tetramer-positive CD8^+^ T cells, splenocytes were obtained from the immunized mice and blocked with clear back (Fc receptor blocking reagent; MBL), stained with PE-conjugated H-2K^b^ OVA tetramer and FITC-conjugated CD8 mAbs (MBL). All stained cells were acquired on FACSCalibur or FACSVerse flow cytometers (BD), and the data were analyzed using FlowJo software (Tree Star). Fluorescence compensation was optimized using cells individually labeled with each fluorochrome-conjugated mAb. Data were obtained from the live population based on cell size- and morphology-based gating, which was used to eliminate cell debris and dead cells.

### *In vivo* Fluorescence Imaging

To visualize the migration of antigen to the dLNs, a fluorescent dye-labeled antigen was prepared by conjugating 1 mg OVA protein with 0.1 mg IRDye800CW fluorescent dye using an IRDye800CW protein labeling kit (LI-COR Bioscience). The concentration of the resulting IRDye800CW-labeled OVA (OVA-IR800) was determined using a BCA protein assay. Hair on the left forepaw and the dorsal skin of C57BL/6 mice were removed by applying depilatory creams (VEET Hair Removal Cream; Reckitt Benckiser Japan) for efficient signal transmission. The mice were anesthetized with 3% isoflurane and intradermally administered 25 μg OVA-IR800 alone or mixed with 400 μg alum, 400 μg γ-PGA, or 800 μg PGA/Alum into the forepaw pad. At 1, 3, 6, 24, and 48 h post-injection, *in vivo* near-infrared (NIR) fluorescent signals from the anesthetized mice were acquired using the *in vivo* Imaging System (IVIS Lumina II; Xenogen Corp.) with excitation at 780 nm and emission at 831 nm at a 0.02 s exposure time. The fluorescent signals of OVA-IR800 in the axillary lymph node were quantitatively analyzed using image analysis ImageJ software (NIH).

### Immunizations and Viral Challenge

Animals were randomly distributed to groups of 3–6 mice. C57BL/6 mice were i.m. immunized with 10 μg OVA in the presence or absence of 400 μg/ml γ-PGA, 400 μg/ml alum, or 800 μg/ml PGA/Alum on days 0, 14, and 28. Spleens, bone marrow cells, and sera were collected on days 14 and 180 after the last immunization. In a separate experiment, mice were i.m. immunized with the pH1N1 split vaccine antigen (A/California/7/2009 NYMC X-179A H1N1), which contained 0.05 μg (for homosubtypic protection) or 0.5 μg (for heterosubtypic protection) hemagglutinin (HA) plus 400 μg γ-PGA, 400 μg alum, or 800 μg PGA/Alum on days 0 and 14. Spleens and sera were collected on day 14 after the last vaccination. Two weeks after the last vaccination, the mice were intranasally challenged with a lethal dose (LD) of influenza viruses, including 50 LD_50_ (equivalent to 30,000 TCID_50_ or 15,000 PFU) A/California/04/09 (pH1N1), 10 LD_50_ (equivalent to 20 TCID_50_ or 200 PFU) A/Puerto Rico/8/34 (H1N1), or 10 LD_50_ (equivalent to 600 TCID_50_ or 300 PFU) H3N2 viruses.

The body weight and survival were monitored for 14 days after the viral challenge. Mice that lost >20% (for homosubtypic viral challenge) or 25% (for heterosubtypic viral challenge) of their body weight were considered to have reached the experimental end point and were sacrificed.

### Antibody-Dependent NK Cell Activation

To examine the ability of the sera Abs of immunized mice to activate NK cells, we coated ELISA plates (Nunc) overnight at 4°C with 10 μg/ml OVA protein or 6 μg/ml influenza HA antigen that contained the stalk domain. The plates were washed with PBS, incubated with heat-inactivated sera (1 h at 56°C) for 2 h at 37°C, and washed with PBS to remove unbound serum Abs. NK cells were isolated from the splenocytes of unimmunized C57BL/6 mice using a NK cell isolation kit (Miltenyi Biotec). The isolated NK cells were dispensed to the ELISA plates (1 × 10^5^ cells/well) and incubated in the presence of PE-conjugated anti-CD107a mAb, 5 μg/ml brefeldin A, and 5 μg/ml monensin for 5 h at 37°C. Finally, the NK cells were harvested, fixed, permeabilized, and intracellularly stained with APC-conjugated anti-IFN-γ using a Cytofix/Cytoperm kit (BD Bioscience).

### ADCC Assay

For experiments using the sera of mice immunized with OVA antigen, B16mOVA cells (target cells) were plated to 96-well U-bottom plates at 8 × 10^3^ cells/well. B16F10 cells were used as a negative control. For experiments using the sera of mice immunized with influenza vaccine antigen, MDCK cells were infected with A/California/04/09 (pH1N1), A/Puerto Rico/8/34 (H1N1), or H3N2 (multiplicity of infection = 1) in serum-free DMEM that contained 100 U/ml penicillin and 100 mg/ml streptomycin for 12 h at 37°C. The virus-infected MDCK cells (target cells) were harvested and plated to 96-well U-bottom plates at 8 × 10^3^ cells/well. The target cells were mixed with both heat-inactivated serum samples (56°C, 1 h) and naïve NK cells and then incubated for 4 h at 37°C. Cytotoxicity was assessed by detection of lactate dehydrogenase (LDH) in culture supernatants using a CytoTox 96 Non-radioactive cytotoxicity assay (Promega).

### Enzyme-Linked Immunospot (ELISPOT) Assay

The frequencies of antigen-specific IFN-γ-producing cells were evaluated using a mouse ELISPOT kit (BD Bioscience), as previously described (18, 19). Briefly, splenocytes were obtained from immunized mice and plated at 5 × 10^5^ cells/well onto purified anti-IFN-γ-coated ELISPOT plates. The cells were stimulated with OVA_257−264_ peptide (0.5 μg/well; Anaspec) or UV-inactivated viruses, including 500 TCID_50_/well of A/California/04/09 (pH1N1), 2,000 TCID_50_/well of A/Puerto Rico/8/34 (H1N1), or 2,000 TCID_50_/well of H3N2 viruses for 3 days. The spot-forming units (SFUs) of IFN-γ-producing cells were counted using an ELISPOT plate reader (Cellular Technology Ltd).

### Hemagglutination-Inhibition (HI) Assay

The serum HI titers against the A/California/04/09 (pH1N1) were determined as previously described (18). HI titers were recorded as the reciprocals of the highest serum dilution at which hemagglutination was prevented.

### Virus Titration in Lungs

On days 3 and 7 post-challenge, mouse lung samples were homogenized in MEM that contained 0.2% BSA at 1 g of lung/ml. The homogenates were centrifuged at 15,000 × *g* for 10 min at 4°C to remove cell debris, and the supernatants were used for the assay. The viral titers were calculated according to the method of Reed and Muench (20) and expressed as log_10_ TCID_50_/ml.

### Statistical Analysis

Data are presented as the means ± standard deviations (SDs) and represent at least three independent experiments. Significant differences between two groups were assessed using the two-tailed Student's *t*-test, and differences among multiple groups were assessed using one-way ANOVA followed by Bonferroni's correction (ANOVA/Bonferroni). The log-rank test was used to analyze survival between two groups. *P* values <0.05 were considered to be statistically significant. All analyses were performed using GraphPad PRISM software (GraphPad).

## Results

### PGA/Alum Complex Shows a High Antigen-Loading Capacity With No Cytotoxicity

**Figure 1:**
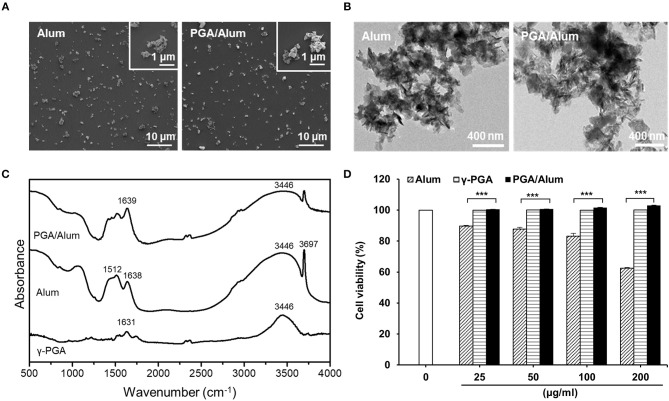
The physiochemical characterization of PGA/Alum. **(A)** SEM and **(B)** TEM images of alum and PGA/Alum (inset images are highly magnified). **(C)** FT-IR spectra of γ-PGA, alum, and PGA/Alum. **(D)** Cytotoxicity of PGA/Alum was evaluated by measuring cell viability of BMDCs exposed to various concentrations of alum, γ-PGA, and PGA/Alum for 24 h. The data are representative of three independent experiments. Statistically significant differences were analyzed via ANOVA/Bonferroni. ***P < 0.001.

To develop a potential adjuvant for future clinical applications, we fabricated PGA/Alum by combining γ-PGA and alum, which is composed of aluminum hydroxide (AH) and magnesium hydroxide (MH). The size and morphology of the fabricated PGA/Alum were measured using dynamic light scattering, TEM, and SEM. Similar to alum, PGA/Alum exhibited an irregular morphology with an average diameter of 1–2 μm (Figures 1A,B), which is consistent with the morphology of alum in a previous report (21). As shown in Table 1, the hydrodynamic diameter of PGA/Alum (1,294.67 ± 13.32 nm) was larger than that of alum (1,066.2 ± 32.01 nm). The polydispersity index was 0.37 ± 0.03 for PGA/Alum and 0.31 ± 0.03 for alum, and was shown in the mid-range of the index value from 0.08 to 0.7 (22). The zeta-potential value of alum was −7.08 ± 0.91 mV, while the value of the PGA/Alum complex was −28.10 ± 1.49 mV, which suggests that γ-PGA was successfully conjugated to alum. To confirm the chemical structures of γ-PGA, alum, and PGA/Alum, we used FT-IR analysis (Figure 1C). In the γ-PGA spectrum, a broad peak at ~3,446 cm^−1^ was attributed to N-H and O-H stretching, while that at 1,631 cm^−1^ was attributed to the C = O stretching of the carbonyl, as previously reported for γ-PGA (23). According to the manufacturer's description, the utilized alum is composed of AH and MH with inactive stabilizers. In the alum spectrum, the peaks at 3,697 and 3,446 cm^−1^ were attributed to the hydroxyl groups of MH and AH, respectively, and those at 1,638 and 1,512 cm^−1^ were attributed to vibrations of the Mg-OH and OH bonds, respectively, in the crystal structure (24–26). The spectrum of PGA/Alum showed distinct peaks that corresponded to alum and γ-PGA at 3,446 and 1,639 cm^−1^, respectively. Based on the results of the FT-IR analysis, we confirmed that γ-PGA and alum successfully formed a complex. We further examined the cytotoxicity of PGA/Alum using BMDCs exposed to alum, γ-PGA, or PGA/Alum for 24 h. As shown in Figure 1D, the treatment of γ-PGA or PGA/Alum did not affect the cell viability, whereas alum dose-dependently decreased the cell viability as previously reported (21). Additionally, immunofluorescent microscopic analysis showed that PGA/Alum was efficiently taken up by BMDCs *in vitro* (Figure S1A). Production of IL-6 and IL-1β rapidly and transiently increased by PGA/Alum in the injection sites by 6 h post-injection but declined almost to basal levels by 24 h post-injection (Figure S1B). We further confirmed the safety of PGA/Alum by measuring body temperature and levels of inflammatory cytokines (IL-6 and TNF-α) in the sera of the vaccinated mice at 6, 24, 48, 72, 96, and 120 h post-vaccination. The PGA/Alum-vaccine group showed no significant change of body temperature compared with PBS or vaccine groups (Figure S2A). The level of serum IL-6 was higher in the PGA/Alum-vaccine and vaccine groups than PBS group at 6 h post-vaccination, but it maintained a basal level at 24 h post-injection. Serum TNF-α level was not changed in PGA/Alum-vaccine, vaccine, and PBS groups (Figure S2B). These results support the safety of PGA/Alum after vaccination. To investigate the antigen encapsulation efficiency, we mixed PGA/Alum with OVA as a model protein antigen and used the BCA assay to measure the amount of loaded OVA. Our results indicated that PGA/Alum loaded significantly more OVA (2.144 ± 0.08 mg/ml) than alum (1.603 ± 0.04 mg/ml) (Table 1). Taken together, our results indicate that the PGA/Alum complex successfully form under the utilized conditions and it shows a high antigen-loading capacity without cytotoxicity.

**Table 1:** Physiochemical characterization and OVA-loaded efficiencies of PGA/Alum.

**Sample**	**Particle size (diameter nm ± SD)**	**Polydispersity index (PDI)**	**Zeta potential (mV)**	**OVA-loaded (mg/ml)**
Alum	1,066.2 ± 32.01	0.31 ± 0.03	−7.08 ± 0.91	1.603 ± 0.04
PGA/Alum	1,294.67 ± 13.32	0.37 ± 0.03	−28.10 ± 1.49	2.144 ± 0.08

### PGA/Alum Significantly Enhances the Activation and Antigen Presentation of DCs *in vitro*

**Figure 2:**
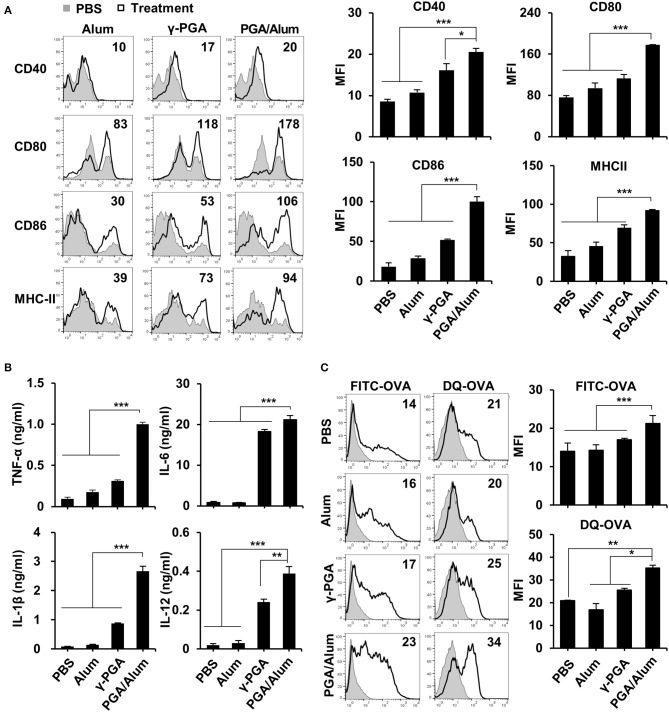
PGA/Alum significantly induces activation and antigen presentation of DCs. Immature BMDCs were stimulated with alum, γ-PGA, and PGA/Alum for 24 h. **(A)** Expression levels of CD40, CD80, CD86, and MHC-II were analyzed by flow cytometry, and **(B)** the levels of cytokines in the culture supernatants were determined by ELISA. **(C)** Immature BMDCs were incubated with either FITC-OVA or DQ-OVA plus PBS or alum, γ-PGA, and PGA/Alum for 1 or 5 h, respectively. Fluorescent intensity was measured via flow cytometry. The numbers in the histograms indicate MFI values. The data are representative of three independent experiments. Statistically significant differences were analyzed via ANOVA/Bonferroni; *P < 0.05, **P < 0.01, and ***P < 0.001.

Because DCs are professional antigen-presenting cells (APCs) that are responsible for the initiation of adaptive immunity (27), we examined the effect of PGA/Alum on DC activation. After immature BMDCs were prepared and stimulated with alum, γ-PGA, or PGA/Alum for 24 h, the levels of pro-inflammatory cytokines and various costimulatory molecules were analyzed via ELISA and flow cytometry, respectively. As shown in Figure 2A, the mean fluorescence intensities (MFIs) of costimulatory molecules (CD40, CD80, and CD86) and MHC class II molecules were substantially increased on the PGA/Alum-exposed BMDCs compared to those treated with alum or γ-PGA. Similarly, PGA/Alum-treated BMDCs produced higher levels of cytokines (TNF-α, IL-6, IL-1β, and IL-12) than those stimulated with alum or γ-PGA alone (*P* < 0.01) (Figure 2B). To investigate the antigen uptake and degradation abilities of these cells, we incubated BMDCs with FITC-OVA or DQ-OVA, which is a self-quenching dye that emits green fluorescence upon the degradation of OVA. Flow cytometry showed that the MFIs of FITC-OVA or DQ-OVA were significantly higher in the PGA/Alum-treated BMDCs than in those treated with PBS, alum, or γ-PGA (Figure 2C), indicating that PGA/Alum substantially increases the antigen-uptake ability of DCs.

Because γ-PGA has been reported to activate DCs via TLR4 signaling (17), we prepared BMDCs from TLR4-defective C3H/HeJ mice and used them to examine whether PGA/Alum-induced DC activation is mediated by TLR4. We observed a significantly lower level of TNF-α in BMDCs from TLR4-defective C3H/HeJ mice than from wild-type C3H/HeN mice following PGA/Alum treatment (Figure S3A). Furthermore, PGA/Alum induced little IκBα phosphorylation in TLR4-defective BMDCs, whereas the phosphorylation in wild-type BMDCs was dose-dependently increased by PGA/Alum (Figure S3B). Taken together, these results indicate that PGA/Alum activates DCs through NF-κB signaling via TLR4.

### PGA/Alum Substantially Increases Antigen Trafficking and Migration of Antigen-Loaded DCs From Injection Sites to Draining Lymph Nodes

**Figure 3:**
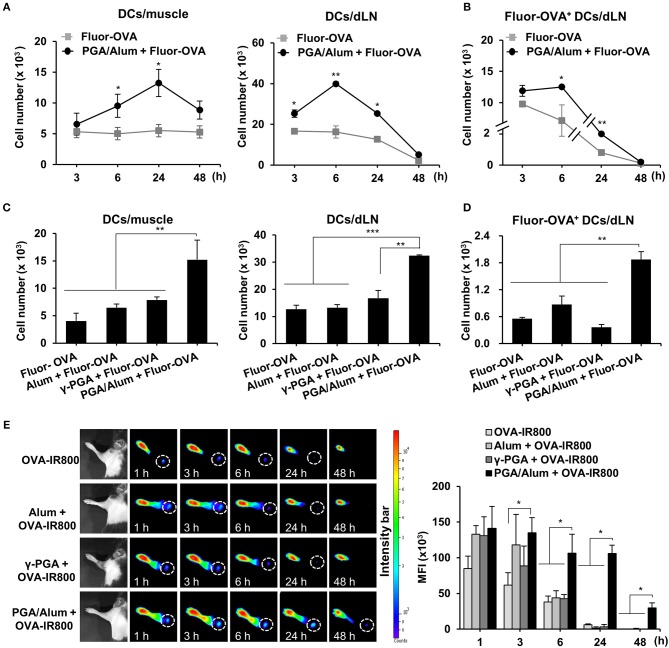
PGA/Alum efficiently increases antigen trafficking from the injection site to dLNs. **(A,B)** C57BL/6 mice (*n* = 3 per group) were i.m. immunized with 5 μg Fluor-OVA mixed with 800 μg PGA/Alum. At 3, 6, 24, and 48 h post-immunization, the number of DCs (gated as CD11c^+^MHC-II^+^ cells) per injected muscle and dLN **(A)** and Fluor-OVA-loaded DCs (gated as Fluor-OVA^+^CD11c^+^MHC-II^+^ cells) per dLN **(B)** were analyzed via flow cytometry. **(C,D)** C57BL/6 mice (*n* = 3 per group) were i.m. immunized with 5 μg Alexa Fluor 647-conjugated OVA alone or combined with 400 μg alum, 400 μg γ-PGA, or 800 μg PGA/Alum. At 24 h post-immunization, the number of DCs per injected muscle and dLN **(C)** and Fluor-OVA-loaded DCs per dLN **(D)** were analyzed via flow cytometry. **(E)** Mice (*n* = 3 per group) were s.c. injected into the right forepaw pad with 25 μg OVA-IR800 alone or combined with 400 μg alum, 400 μg γ-PGA, or 800 μg PGA/Alum. At the indicated time points, *in vivo* NIR fluorescence signals were acquired using IVIS. Fluorescent intensities of each region of interest were quantitatively measured using ImageJ software (circle: axillary lymph node). The data are representative of three independent experiments. Statistically significant differences were identified via *t*-test; *P < 0.05, **P < 0.01, ***P < 0.001. N.D, not-detected.

The recruitment of DCs to the antigen injection site and the migration of antigen-loaded DCs to dLNs are vital steps in the induction of adaptive immunity and are important to the efficacy of a vaccine. Accordingly, we used Alexa Fluor 647-conjugated OVA (Fluor-OVA) to examine the effect of PGA/Alum on the recruitment of DCs and the migration of antigen-loaded DCs. Mice were i.m. injected with Fluor-OVA mixed with or without PGA/Alum, and flow cytometry was used to enumerate the DCs in injected muscle tissues and dLNs. Compared with the Fluor-OVA group, the number of DCs (CD11c^+^MHC-II^+^) was significantly increased in the muscle tissues of the PGA/Alum-Fluor-OVA group at 6 and 24 h post-injection (Figure 3A). Notably, we observed ~2-fold more DCs in the muscle tissues of the PGA/Alum-Fluor-OVA group (13.3 ± 2.2 × 10^3^ cells/muscle) than in the Fluor-OVA group (5.5 ± 0.7 × 10^3^ cells/muscle) at 24 h post-injection. Importantly, the number of antigen-loaded DCs (Fluor-OVA^+^CD11c^+^MHC-II^+^) was increased in the dLNs of the PGA/Alum-Fluor-OVA group compared to the Fluor-OVA group, particularly at 6 h post-injection (12.5 ± 0.1 × 10^3^ cells/LN for the PGA/Alum-Fluor-OVA group and 7.1 ± 2.5 × 10^3^ cells/LN for the Fluor-OVA group) (*P* < 0.05) (Figure 3B). The fluorescent intensity of Fluor-OVA and the accumulation of CD11c^+^MHC-II^+^ cells increased in both the injected muscle tissues and dLNs of the PGA/Alum-Fluor-OVA group at 6 h post-injection (Figure S4A). We also observed increased numbers of neutrophils and monocytes in the injected muscles of the PGA/Alum-Fluor-OVA group compared with the Fluor-OVA group at 3, 6, and 24 h post-injection (Figure S4B). Compared to other groups, the PGA/Alum-Fluor-OVA group had significantly higher DC numbers in injected muscle tissues and dLNs (Figure 3C) and higher antigen-loaded DCs numbers in dLNs (Figure 3D) at 24 h post-injection.

To assess the antigen delivery activity of PGA/Alum, we performed *in vivo* imaging of the antigen trafficking by PGA/Alum from the injection site to the dLNs using OVA with an NIR fluorescence imaging system. IRDye800-labeled OVA (OVA-IR800) alone or combined with alum, γ-PGA, or PGA/Alum were subcutaneously (s.c.) injected into the right forepaw pad of C57BL/6 mice, and *in vivo* fluorescent signals were observed at 1, 3, 6, 24, and 48 h post-injection. As shown in Figure 3E, the fluorescent intensities were significantly higher in the dLNs of the mice treated with PGA/Alum-mixed OVA-IR800 than in those of the mice exposed to OVA-IR800 alone or mixed with alum or γ-PGA at 6, 24, and 48 h post-injection. Notably, the sustained fluorescent signal was observed in the dLNs of the PGA/Alum-mixed OVA-IR800-treated mice until 48 h post-administration, but not in other groups, which implies that PGA/Alum acts as an efficient antigen carrier.

As chemokines modulate the migration of immune cells to dLNs (28–30), levels of various chemokines (MIP-1α, MIP-1β, and MCP-1) were substantially enhanced in the homogenates from the muscle tissues of the PGA/Alum-OVA group, but not in those of the OVA group (*P* < 0.001) (Figure S5A). Increased chemokine levels were also observed in the dLN homogenates of the PGA/Alum-OVA group, but not in those of the OVA group. We additionally examined the expression of CCR7 responsible for migration of DCs from the antigen exposure site to dLNs (31). As expected, the expression of CCR7 was nearly 3-fold higher on the PGA/Alum-exposed DCs than on those exposed to alum or γ-PGA (Figure S5B). Also, the mice administered PGA/Alum-mixed Fluor-OVA had significantly higher expression of CCR7 on the DCs in the dLNs than other groups (Figure S5C). Collectively, these results indicate that PGA/Alum enhances the recruitment of DCs to injection sites and the migration of antigen-loaded DCs to dLNs through increases of chemokine production and CCR7 expression.

### PGA/Alum Enhances OVA-Specific Humoral and Cellular Immune Responses

**Figure 4:**
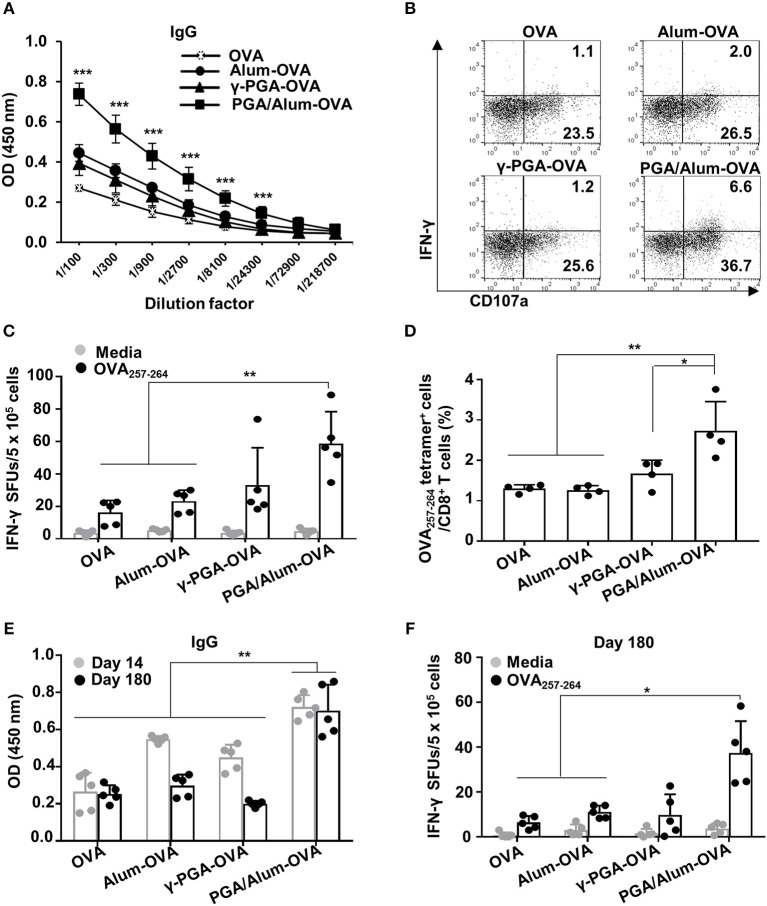
PGA/Alum enhances OVA-specific humoral and cellular immune responses. C57BL/6 mice (*n* = 5 per group) were i.m. immunized with 10 μg OVA protein combined with 400 μg alum, 400 μg γ-PGA, or 800 μg PGA/Alum on days 0, 14, and 28. Fourteen **(A–D)** and 180 **(E,F)** days after the last immunization, sera and splenocytes were obtained from the immunized mice. **(A)** Serum levels of OVA-specific IgG were measured via ELISA. **(B)** OVA-coated plates were incubated with heat-inactivated serum samples (1 h at 56°C), washed, and then further incubated for 5 h with naïve NK cells in the presence of PE-conjugated anti-CD107a Ab, monensin, and brefeldin A. The cells were fixed, permeabilized, and stained with APC-conjugated anti-IFN-γ Ab. Activation of NK cells was assessed by flow cytometry. **(C)** Splenocytes were stimulated with OVA_257−264_ peptide for 3 days, and the number of OVA_257−264_-specific IFN-γ spot forming units (SFUs) was determined by an ELISPOT assay. **(D)** Frequency of OVA_257−264_ tetramer^+^ CD8^+^ T cells was determined in the splenocytes using flow cytometry. **(E)** The level of OVA-specific IgG at days 14 and 180 post-immunization and **(F)** the number of OVA_257−264_-specific IFN-γ SFUs on day 180 were determined by ELISA and an ELISPOT assay, respectively. Data are representative of three independent experiments with similar results. Statistically significant differences were identified via ANOVA/Bonferroni; *P < 0.05, **P < 0.01, and ***P < 0.001.

To evaluate the effect of PGA/Alum on antigen-specific humoral and cellular immunity, we i.m. injected mice with OVA mixed with alum (alum-OVA), γ-PGA (γ-PGA-OVA), or PGA/Alum (PGA/Alum-OVA) on days 0, 14, and 28. Two weeks after the last immunization, ELISA was employed to measure the levels of OVA-specific Abs in the sera of the immunized mice. Our results showed that the levels of IgG, IgG1, and IgG2b significantly increased in the sera of the PGA/Alum-OVA group compared with those of the OVA, alum-OVA, and γ-PGA-OVA groups (*P* < 0.001) (Figure 4A; Figure S6). This finding indicates that PGA/Alum increases antigen-specific humoral immunity.

We subsequently examined the ADCC of natural killer (NK) cells, which is crucial for eliminating Ab-bound target cells (e.g., virus-infected cells) (32, 33). To investigate the effect of PGA/Alum on the ADCC activity of NK cells, we examined the CD107a expression and the IFN-γ production of NK cells, as well as target cell cytolysis using B16mOVA cells (OVA-expressed B16F10 cells). The activation of NK cells was robustly enhanced by co-culture with a mixture of B16mOVA cells and the sera of the PGA/Alum-OVA group (5.5 ± 1.2% for IFN-γ^+^CD107a^+^ and 39.6 ± 4.6% for CD107a^+^) compared with the OVA (1.2 ± 0.1% for IFN-γ^+^CD107a^+^ and 25.9 ± 1.6% for CD107a^+^), γ-PGA-OVA (1.2 ± 0.3% for IFN-γ^+^CD107a^+^ and 25.8 ± 2.4% for CD107a^+^), and alum-OVA (2.1 ± 0.1% for IFN-γ^+^CD107a^+^ and 27.3 ± 1.9% for CD107a^+^) groups (Figure 4B; Figure S7A). The percentage of cytotoxicity was also higher in the PGA/Alum-OVA group (60 ± 5%) than in the γ-PGA-OVA (50 ± 4%), alum-OVA (50 ± 8%), or OVA (41 ± 3%) groups (Figure S7B). In addition, we investigated whether PGA/Alum enhances cellular immune responses specific to OVA_257−264_ peptides which are MHC class I-restricted peptide epitope of OVA. ELISPOT assay revealed that the number of OVA_257−264_-specific IFN-γ-secreting cells was significantly higher in the PGA/Alum-OVA group than in the OVA or alum-OVA groups (*P* < 0.01) (Figure 4C). Moreover, flow cytometry showed that the frequency of OVA_257−264_ tetramer-positive CD8^+^ T cells was significantly increased in PGA/Alum-OVA compared to other groups (*P* < 0.01) (Figure 4D). These findings indicate that PGA/Alum enhances antigen-specific cellular immune responses, such as ADCC and CTL activities.

As immunological memory is a critical goal for effective vaccination, we examined the long-term immunity of mice treated with OVA, alum-OVA, γ-PGA-OVA, or PGA/Alum-OVA. As shown in Figure 4E, on day 180 after the final immunization, the PGA/Alum-OVA group showed a significantly higher IgG level than the other groups (*P* < 0.01). Notably, the increased IgG level of the PGA/Alum-OVA group was similar on days 14 and 180 after the final immunization, whereas those of the alum-OVA and γ-PGA-OVA groups had decreased to the baseline level by day 180 after the final immunization. The frequency of OVA_257−264_-specific IFN-γ-secreting cells was also higher in the PGA/Alum-OVA group than in the groups treated with OVA or alum-OVA (*P* < 0.01) (Figure 4F). The PGA/Alum-OVA group also exhibited higher IgG1 and IgG2b levels (Figure S8A), greater percentages of both plasma cells and memory B cells (Figure S8B), more ADCC activity (Figure S8C), and a higher frequency of OVA_323−339_ peptide-specific IL-4-secreting cells (Figure S8D) on day 180 after the final immunization. Taken together, these results demonstrate that PGA/Alum could be a potential adjuvant capable of enhancing humoral and cellular immunity and inducing persistent long-term immunity.

### PGA/Alum Substantially Enhances the Protective Efficacy of Influenza Vaccine Antigen

**Figure 5:**
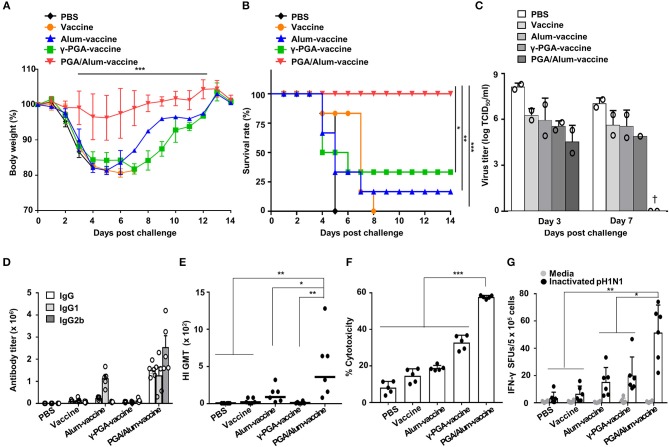
PGA/Alum improves the protective efficacy of influenza vaccine antigen. C57BL/6 mice (*n* = 6 per group) were i.m. immunized with the 0.05 μg pH1N1 split vaccine antigen combined with 400 μg alum, 400 μg γ-PGA, or 800 μg PGA/Alum on days 0 and 14. Two weeks after the final administration, the mice were i.n. challenged with 50 LD_50_ pH1N1 virus. **(A)** Body weight changes and **(B)** survival rates were monitored for up to 14 days post-infection (dpi). Each data point represents an average percentage. Statistically significant differences were identified via the log-rank test. **(C)** Lung homogenates of each group were obtained at 3 and 7 dpi. Viral titers were determined using the pooled lung homogenates in duplicate and are expressed as log_10_TCID_50_/ml (†, virus clearance). **(D)** Sera (*n* = 5 per group) were collected before the viral challenge, and endpoint titers of vaccine-specific Abs are expressed as the mean ± SD. **(E)** Serum HI titers against a pH1N1 virus were measured. The lines indicate geometric means, and negative titers were assigned a value of 5 for calculation. **(F)** The pH1N1-infected MDCK cells were incubated with naïve NK cells in the presence of the sera from immunized mice, and cytotoxicity was assessed by LDH assay. **(G)** The number of influenza virus antigen-specific IFN-γ-secreting splenocytes was determined via ELISPOT assays. Data are representative of at least three independent experiments. Statistically significant differences were identified by ANOVA/Bonferroni; *P < 0.05, **P < 0.01, and ***P < 0.001.

To evaluate whether PGA/Alum improves the protective efficacy of a vaccine antigen, we investigated the effect of PGA/Alum on the immunogenicity and efficacy of pH1N1 split vaccine antigen. Mice were i.m. immunized with the pH1N1 split vaccine antigen (A/California/7/2009 NYMC X-179A H1N1) mixed with PGA/Alum (PGA/Alum-vaccine), alum (alum-vaccine), or γ-PGA (γ-PGA-vaccine) on days 0 and 14. The mice immunized with vaccine antigen alone or PBS were used as negative controls. Fourteen days after the final immunization, the immunized mice were intranasally (i.n.) challenged with a lethal dose (50 LD_50_) of pH1N1 virus (A/California/04/09). As shown in Figures 5A,B, the mice of the PGA/Alum-vaccine group showed 100% survival without considerable body weight loss for 14 days after this viral challenge. In contrast, the mice immunized with alum-vaccine and γ-PGA-vaccine showed severe body weight loss and were only partially protected, exhibiting survival rates of 16.7 and 33.3%, respectively. The mice immunized with PBS or vaccine alone had 0% survival. As viral clearance from the infected lung is a crucial indicator of the protective efficacy of a vaccine, we determined viral titers in lung homogenates on days 3 and 7 post-infection. As expected, the PGA/Alum-vaccine group exhibited viral clearance on day 7 post-challenge, whereas the other groups continued to exhibit high viral titers at this point (Figure 5C). Our findings indicate that PGA/Alum substantially enhances the protection efficacy of the pH1N1 split vaccine antigen against pH1N1 virus challenge.

To confirm the adjuvant effect of PGA/Alum for the influenza vaccine, we examined IgG titers, hemagglutination-inhibition (HI) titers, ADCC, and CTL activities. As shown in Figure 5D, the titers of influenza antigen-specific IgG, IgG1, and IgG2b were higher in the sera obtained from the mice of the PGA/Alum-vaccine group than those obtained from the other groups. The HI titers were also substantially increased in the sera of the mice from the PGA/Alum-vaccine group (359 ± 180 geometric mean titer [GMT]) compared to those of the other groups (90 ± 45 GMT for alum-vaccine, 9 ± 6 GMT for γ-PGA-vaccine, 20 ± 14 GMT for vaccine, and 1 ± GMT for PBS) (Figure 5E), which indicates that PGA/Alum confers neutralizing humoral immunity. Next, when ADCC activity was analyzed by measuring lysis of pH1N1-infected MDCK cells through co-culture of NK cells and the sera Abs of immunized mice, the sera Abs of PGA/Alum-vaccine group had significantly higher ADCC activities (58.2 ± 0.5%) compared with those of the other groups (18.5 ± 0.2% for alum-vaccine, 32.8 ± 3.6% for γ-PGA-vaccine, 14.7 ± 3.5% for vaccine, and 8.2 ± 3.1% for PBS) (Figure 5F). Moreover, ELISPOT assay showed that the mice of the PGA/Alum-vaccine group yielded significantly more pH1N1 virus-specific IFN-γ-secreting cells than the other groups (*P* < 0.05) (Figure 5G). The percentages of IFN-γ-secreting CD4^+^ and CD8^+^ T cells were also higher in the PGA/Alum group than in the other groups (Figure S9). Taken together, our results suggest that PGA/Alum enhances the protective efficacy of the influenza vaccine antigen by modulating influenza antigen-specific humoral and cellular immunity.

### PGA/Alum Substantially Improves the Cross-Reactive Immunity of Influenza Vaccine Antigen

**Figure 6:**
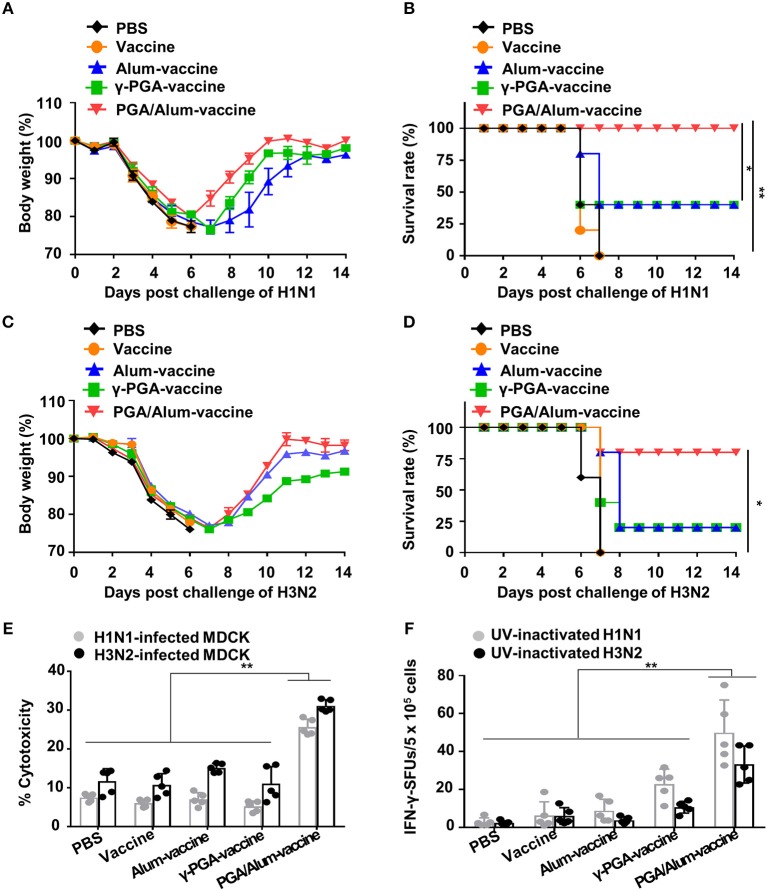
PGA/Alum enhances cross-protective efficacy of pH1N1 vaccine antigen. C57BL/6 mice (*n* = 5 per group) were vaccinated i.m. with 0.5 μg the pH1N1 split vaccine antigen together with 400 μg alum, 400 μg γ-PGA, or 800 μg PGA/Alum on days 0 and 14. Two week after the last immunization, the mice were i.n. challenged with 10 LD_50_ H1N1 virus (A/Puerto Rico/8/1934) **(A,B)** or 10 LD_50_ H3N2 viruses **(C,D)**. Body weight and survival rates were monitored for 14 days. **(E,F)** Before viral challenge, sera and splenocytes were harvested from the immunized mice. **(E)** ADCC activity was determined by measuring lysis of H1N1- or H3N2-infected MDCK cells by co-culture of sera from the vaccinated mice and naïve NK cells. **(F)** Splenocytes were stimulated with UV-inactivated H1N1 or UV-inactivated H3N2 for 3 days, and the number of IFN-γ^+^ SFUs was determined by an ELISPOT assay. Statistically significant differences were identified by one-way ANOVA/Bonferroni or log-rank test (for survival); *P < 0.05 and **P < 0.01.

Given our observations that PGA/Alum increased the ADCC and CTL activities responsible for cross-reactivity against heterologous influenza virus, we speculated that PGA/Alum might enhance the cross-protective efficacy of pH1N1 vaccine antigen. To evaluate the impact of PGA/Alum on this cross-reactivity, mice were immunized the pH1N1 split vaccine antigen mixed with alum, γ-PGA, or PGA/Alum and then challenged with A/Puerto Rico/8/34 (H1N1). Body weight decreased similarly across all groups until 6 days after the viral challenge, but was recovered faster by the PGA/Alum-vaccine group compared to the other groups (Figure 6A). Importantly, all mice of the PGA/Alum-vaccine group showed 100% survival, whereas only partial survival (40%) was observed in the alum-vaccine and γ-PGA-vaccine groups (Figure 6B). To ascertain the effect of PGA/Alum on cross-protective immunity, we challenged the vaccinated mice with heterosubtypic influenza A virus, H3N2. Mice of the PGA/Alum-vaccine group efficiently recovered the body weight lost following viral challenge and showed 80% survival. In contrast, only 20% of mice in the alum-vaccine or γ-PGA-vaccine groups survived, and no survival was seen in the PBS or vaccine-alone groups (Figures 6C,D).

To better understand the mechanisms underlying PGA/Alum-enhanced cross-reactive protection, we examined cross-reactive ADCC and CTL activities. Our ADCC assay revealed that the cytolysis of heterologous influenza virus (H1N1 or H3N2)-infected MDCK cells was significantly increased by co-culture with naïve NK cells and serum Abs obtained from the PGA/Alum-vaccine group (25.6 ± 1.9% for H1N1-infected MDCK cells and 31.1 ± 1.3% for H3N2-infected MDCK cells), but not serum Abs obtained from mice exposed to alum-vaccine (6.9 ± 1.3% and 15.1 ± 1%, respectively), γ-PGA-vaccine (5.2 ± 1.2 and 11.1 ± 3.5%, respectively), vaccine alone (6.0 ± 0.8 and 10.7 ± 2.9%, respectively), or PBS (7.5 ± 0.9 and 11.8 ± 2.9%, respectively) (Figure 6E). To clearly elucidate Ab-mediated contribution of PGA/Alum-enhanced heterosubtypic cross-protection, we further performed *in vivo* protection assay as previously described (34, 35). Heat-inactivated sera from the immunized mice were mixed with H3N2 virus, and then the mixture was i.n. challenged to Balb/c mice. As shown in Figure S10, at day 14 post-infection, mice exposed to the mixture of H3N2 virus and PGA/Alum-vaccine-immunized sera had 20% survival rate, whereas all mice of other groups died (0% survival rate), indicating that PGA/Alum-enhanced Ab production may partially contribute to heterosubtypic cross-protection. As the ADCC-mediating Abs are thought to recognize the highly conserved stalk domain of hemagglutinin (HA) on the influenza virus, thereby leading to cross-protection (36), we tested ADCC-mediated NK cell activation by incubating the serum Abs with the HA stalk protein. Our results revealed that HA stalk protein-preincubated serum Abs from the PGA/Alum-vaccine group significantly increased the percentages of IFN-γ^+^ NK and CD107^+^IFN-γ^+^ NK cells (Figure S11), indicating that the PGA/Alum-induced Abs effectively bind to the HA stalk domain and subsequently activate NK cells. Additionally, we performed ELISAs to measure antibodies against the HA stalk using a recombinant HA stalk protein from A/Puerto Rico/8/1934 (H1N1) which share 94% identity to A/California/04/09 (pH1N1) and 50% identity to A/Hong Kong/1/68 (H3N2) virus. HA stalk-specific IgG level was significantly higher in the sera from the PGA/Alum-vaccine group than those in other groups, although the pH1N1 vaccine alone group very little elicited H1 HA stalk-specific IgG level (Figure S12A). We further tested H3 ELISA using a recombinant HA1 protein of H3N2 virus to investigate whether cross-reactive neutralizing Abs contribute to cross-protection against H3N2 virus. None of the pH1N1 vaccine antigen-immunized groups elicited H3 HA1-specific IgG titers (Figure S12B). Moreover, the sera Abs from all groups had no cross HI reactivity against heterosubtypic H3 virus (Figure S12C). These results suggest that PGA/Alum elicits HA stalk Abs but not cross-reactive neutralizing Abs. Furthermore, an ELISPOT assay revealed that PGA/Alum increased cross-reactive CTL activity. As shown in Figure 6F, the number of H1N1-specific IFN-γ-secreting splenocytes was at least 2-fold higher in the PGA/Alum-vaccine group (50 ± 17 SFUs) compared to the other groups (23 ± 8 SFUs for γ-PGA-vaccine, 9 ± 6 SFUs for alum-vaccine, 6 ± 7 SFUs for vaccine alone, and 3 ± 2 SFUs for PBS). Moreover, the number of H3N2-specific IFN-γ-secreting cells was at least 3-fold higher in the PGA/Alum-vaccine group (33 ± 10 SFUs) than in the other groups (11 ± 3 SFUs for γ-PGA-vaccine, 4 ± 2 SFUs for alum-vaccine, 6 ± 4 SFUs for vaccine alone, and 2 ± 1 SFUs for PBS) (*P* < 0.01). Collectively, these findings demonstrate that PGA/Alum could enhance cross-protection by improving cross-reactive ADCC and CTL activities.

## Discussion

**Figure 7:**
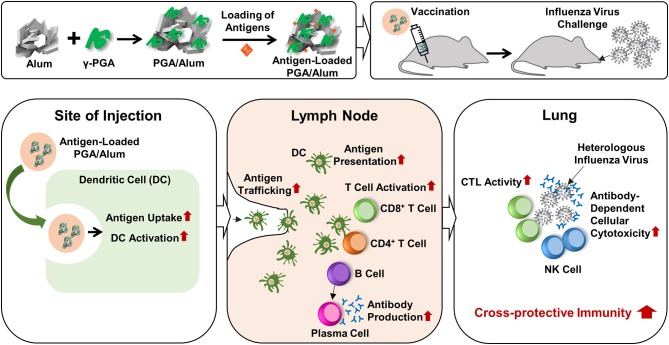
Schematic illustration of the fabrication of PGA/Alum and its action mechanism. PGA/Alum induces the innate immunity such as antigen uptake and delivery by DCs to lymph nodes, thereby leading to the enhanced antigen-specific cellular and humoral immunities. Consequently, PGA/Alum improves the cross-reactive immunity of influenza vaccine antigens against heterologous influenza virus, with increases of CTL and ADCC activities.

Currently, researchers are seeking to develop new adjuvants that increase vaccine-induced protection against infectious diseases. The use of TLR agonists as vaccine adjuvants is considered a promising means to improve vaccine efficacy, because TLR4 activates innate immune responses and subsequently augments adaptive immune responses by enhancing Th1-biased responses (14, 15). The combination of a TLR4 agonist with a Th2 adjuvant (e.g., alum) could be a beneficial strategy for tailoring immune responses through synergistic effects. The value of this strategy was emphasized by the recent approval for human use of AS04, which comprises MPL (a TLR4 agonist) absorbed on alum (37, 38). However, the purification of MPL requires extensive chemical modification of biologically derived LPS. There is a large batch-to-batch variability, the cost is prohibitive, and there are safety concerns. To address these limitations, we set out to replace MPL with the safe and cost-effective biomaterial, γ-PGA, to generate the new combination of PGA/Alum and demonstrated its efficacy as an adjuvant, as summarized in Figure 7. We propose that the synergistic effect of PGA/Alum is mediated mainly by γ-PGA-induced innate immune activity plus alum-induced depot effect. γ-PGA activates innate immune responses including increases of costimulatory molecule expression and cytokine production of APCs. Concomitantly, alum induces vaccine antigen depots capable of enhancing antigen presentation by activating APCs. Also, antigen loading capacity of PGA/Alum may be enhanced by biopolymeric property of γ-PGA and antigen absorption capacity of alum. The combined effect of γ-PGA and alum could robustly provoke Th1 immune responses to enhance CTL activity and highly increase humoral immune responses, thereby leading to improved vaccine efficacy.

An ideal vaccine adjuvant should have a broad-spectrum of safety. We observed that PGA/Alum had very little cytotoxic effect on cells *in vitro*, although alum itself induced cytotoxicity. We speculate that the combination of γ-PGA with alum may be able to block the cytotoxicity of alum. It has been reported that immunization of MF59-adjuvanted influenza vaccine induced adverse reaction (e.g., redness and swelling at the injection site) compared to unadjuvanted vaccine in children (39, 40). By visual observation, administration of PGA/Alum induced no redness and swelling at the injection site (data not shown). Especially, no significant changes of body temperature as well as inflammatory cytokines were observed in the sera of the vaccinated mice. These findings suggest that PGA/Alum may be safe to use. In addition, γ-PGA can be produced on an industrial scale without complex requirements, and thus does not have the limitations associated with the complicated process required to manufacture MPL. A side-by-side comparison of adjuvants including MF59 and AS04 is important to elucidate the adjuvant effect of PGA/Alum. Unfortunately, we cannot compare the adjuvant effect of AS04 and PGA/Alum, because AS04 is unavailable for research use. In the case of MF59, we made squalene-based oil-in-water nano-emulsion with a formulation similar to MF59 (MF59-like adjuvant). In influenza vaccine experiments, PC nanogel [γ-PGA/chitosan nanogel adjuvant published in (18)] had similar protective efficacy with MF59-like adjuvant but lower than that of PGA/Alum (unpublished data). We also observed that MF59-like adjuvant did not induce cell-mediated immunity against the influenza vaccine antigen by IFN-γ ELISPOT assay but PGA/Alum robustly enhanced cell-mediated immunity (unpublished data). Therefore, we imply that PGA/Alum could act as a more potent adjuvant than MF59.

Activated DCs critically contribute to antigen processing and presentation. The activation of DCs and their delivery of antigen to LNs are crucial for the ability of a vaccine to effectively initiate innate and adaptive immune responses (41). Our results showed that PGA/Alum induces DC activation through TLR4 signaling, as assessed by increases in the levels of costimulatory molecules and pro-inflammatory cytokines, as well as enhancement of antigen processing. PGA/Alum robustly enhanced both antigen trafficking and the migration of antigen-loaded DCs from the injection sites to dLNs, which is consistent with previous findings obtained with the MPL-based adjuvants, AS04 and AS01 (37, 38). Our immunofluorescent microscopic analysis further revealed that PGA/Alum increased antigen accumulation in the subcapsular, cortical, and medullary sinus regions of dLNs, indicating that antigen presentation in LNs could be facilitated by PGA/Alum. Circulating immature DCs reach inflamed tissues by following the chemoattractant gradient to uptake antigens and then migrate to dLNs to initiate adaptive immunity (29). Thus, it is relevant that PGA/Alum was found to robustly enhance not only the recruitment of DCs to injection sites and dLNs but also the migration of antigen-loaded DCs to dLNs. Consistent with previous studies in which mice were injected with other adjuvants, including AS04, AS01, and AS03 (37, 38, 42), PGA/Alum increased the levels of chemokines (MIP-1α, MIP-1β, and MCP-1) at injection sites and dLNs. We also found that the expression of CCR7 on DCs, which is responsible for their migration to LNs, was increased by PGA/Alum. Notably, this has not been reported for AS04. Thus, the ability of PGA/Alum to enhance DC migration by increasing chemokine levels and CCR7 expression might provide insight into the action mechanisms of this vaccine adjuvant. Together, our findings demonstrate that PGA/Alum can act as a potent adjuvant capable of activating innate immune responses, including DC activation, DC migration, and antigen trafficking.

Our proof-of-concept experiments demonstrated that PGA/Alum efficiently enhances the humoral and cellular immune responses specific to both OVA (a model antigen) and the influenza vaccine antigen (a vaccine antigen of a representative infectious disease). Importantly, we found that PGA/Alum significantly increased ADCC, which has recently been shown to induce effective protection against various viruses, including Ebola (9), human immunodeficiency virus (43, 44), Epstein-Barr virus (45), and influenza viruses (8, 32). Our results from an ADCC assay performed using mouse serum revealed that PGA/Alum enhanced ADCC activity. As ADCC is known to be initiated by the IgG2 subclass in mice (32), it is notable that we observed a significant induction of IgG2b in the sera of mice immunized with the antigen mixed with PGA/Alum. Because γ-PGA induces Th1 responses (17, 46–48), which are associated with the induction of IgG2b (49), our results suggest that γ-PGA acts synergistically with alum in PGA/Alum to elevate the production of IgG2b and thereby enhance ADCC.

Influenza A viruses exist as several subtypes, and new viruses can emerge due to point mutations (e.g., antigenic drift) or genetic reassortments between different viral subtypes (e.g., antigen shift), potentially leading to influenza epidemics and pandemics (50). The cross-reactivity of an influenza vaccine is essential for its ability to broadly protect against antigenically drifted influenza viruses. Our present results reveal that PGA/Alum enhanced the protective efficacy of the influenza pH1N1 vaccine against homologous virus. Importantly, PGA/Alum-adjuvanted pH1N1 vaccine exhibited improved cross-protection against both heterologous influenza virus (e.g., H1N1) and heterosubtypic virus (e.g., H3N2). In contrast, cross-protection was not observed in mice immunized with alum- or γ-PGA-adjuvanted pH1N1 vaccine. As cross-reactive immunity is primarily mediated by CTLs, which recognize broadly conserved epitopes shared by influenza A virus subtypes (50), our results indicate that PGA/Alum-adjuvanted pH1N1 vaccine drastically enhanced cross-reactive CTL activities against H1N1 and H3N2 viruses. In this experiment, we used a reassortant H3N2 virus carrying HA and NA genes from the A/Hong Kong/1/68 and internal genes from A/Puerto Rico/8/34. Cell mediated immunity against conservation of internal proteins may contribute to PGA/Alum-enhanced heterosubtypic protection. In addition, PGA/Alum enhanced ADCC, which is considered to be the crucial function of non-neutralizing Abs in cross-reactive immunity, and pH1N1 vaccine-PGA/Alum-induced sera Abs had cross-reactive ADCC activity against H1N1 and H3N2 viruses. Consistent with previous reports showing that ADCC-mediating Abs bind to the conserved HA stalk domain of the influenza virus (10, 51), PGA/Alum increased the level of the HA stalk Abs but not cross-reactive neutralizing Abs of heterologous virus (Figure S12). *In vivo* cross-protection assay conferred that PGA/Alum-enhanced heterosubtypic cross-protection may be mainly cell-mediated and partially by humoral immunity, similar with a previous report (Figure S10) (52). Thus, PGA/Alum might help improve the protective efficacy and cross-protection of the influenza vaccine, and its use as an adjuvant could help resolve the limitations of the current influenza vaccines. Given that ADCC and CTL activities have been suggested to contribute to protecting elderly individuals against influenza virus infection (53, 54), our results suggest that PGA/Alum could be used as a vaccine adjuvant for older people with dysregulated immune responses. Taken together, our findings indicate that PGA/Alum may be a promising candidate as a vaccine adjuvant for preventing diseases caused by influenza viruses and other infectious agents.

## Data Availability

The raw data supporting the conclusions of this manuscript will be made available by the authors, without undue reservation, to any qualified researcher.

## Ethics Statement

All animal experiments were reviewed and approved by the Institutional Animal Care and Use Committee (IACUC) of the KRIBB and were performed according to the Guidelines for Animal Experiments of the KRIBB.

## Author Contributions

HP designed and supervised the experiments, analyzed results, and wrote the manuscript. QN, CK, JK, WL, and JY performed the experiments. QN, CK, and JY analyzed the experiments and drafted the manuscript. MS contributed to produce γ-PGA and discuss the results. WL and JJ contributed to analyze chemical characterization of the PGA/Alum.

### Conflict of Interest Statement

The authors declare that the research was conducted in the absence of any commercial or financial relationships that could be construed as a potential conflict of interest.
