# Supplementary material for: Poly-γ-Glutamic Acid Complexed With Alum Induces Cross-Protective Immunity of Pandemic H1N1 Vaccine
Source: Front Immunol. 2019 Jul 11;10:1604. doi: 10.3389/fimmu.2019.01604 (PMC6637289; doi:10.3389/fimmu.2019.01604)
Supplement: Supplementary file 1 [file Data_Sheet_1.pdf]

Supplementary information

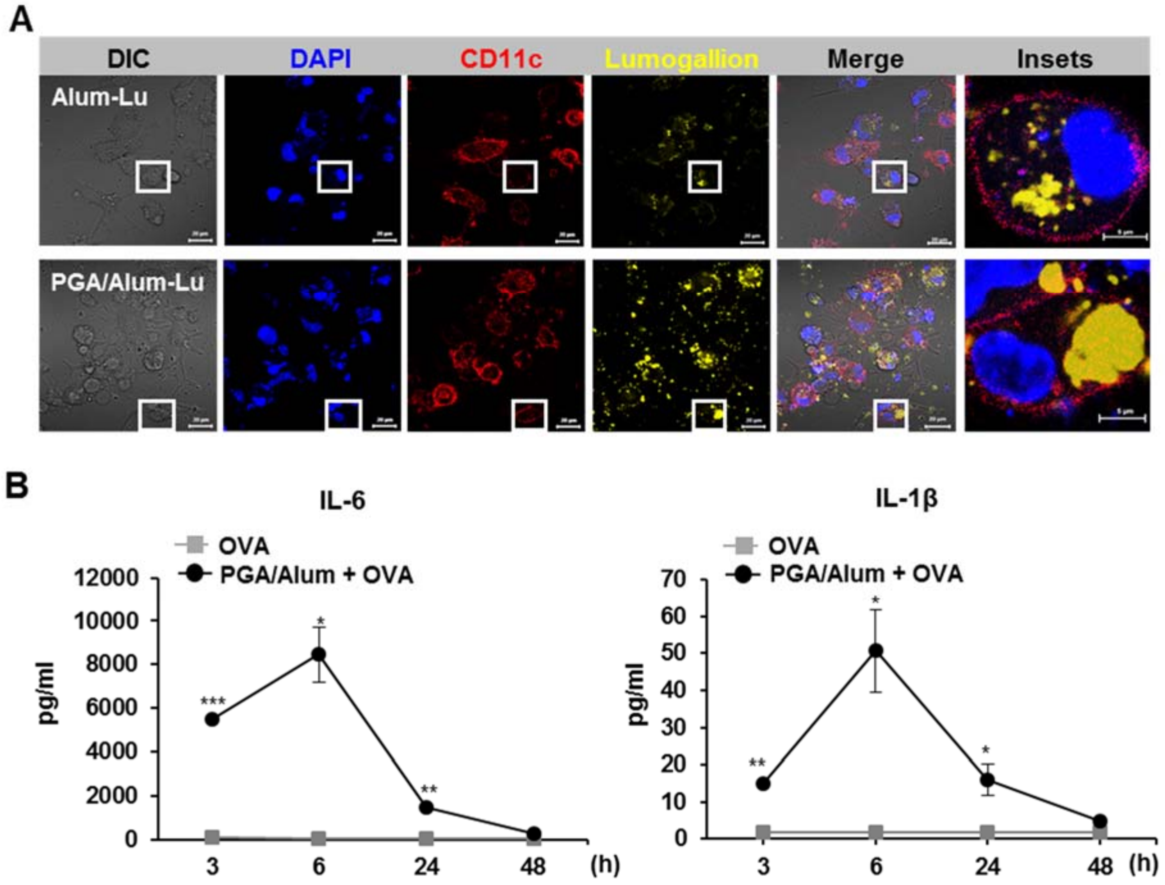

Supplementary Figure 1. PGA/Alum is efficiently taken up by BMDC *in vitro* and induces a rapid and transient local inflammatory response *in vivo*. (A) Fluorescent dyes-conjugated alum and PGA/Alum were prepared by labeling with 100 μM lumogallion, a fluorimetric reagent for the detection of alum. BMDCs were incubated with lumogallion-labeled alum (alum-Lu) at 100 μg/ml or lumogallion-labeled PGA/Alum (PGA/Alum-Lu) at 200 μg/ml for 4 h at 37ºC. The cells were then stained with anti-CD11c antibody (red). Nuclei were counterstained with DAPI (blue). Signal intensities of lumogallion (yellow) were observed to evaluate cellular uptake of PGA/Alum by BMDCs via immunofluorescent microscopic analysis (scale bar 20 μm, and 5 μm for insets). (B) C57BL/6 mice (n = 3 per group) were i.m immunized in tibialis anterior with 5 μg OVA alone or combined with 800 μg PGA/Alum. At 3, 6, 24, and 48 h post-immunization, levels of cytokines were determined in the tissue homogenates of the injected muscles using a Legendplex immunoassay kit. The data are presented as a mean ± SD, and statistically significant differences were analyzed by student t-test; *$P < 0.05$, **$P < 0.01$, and ***$P < 0.001$.

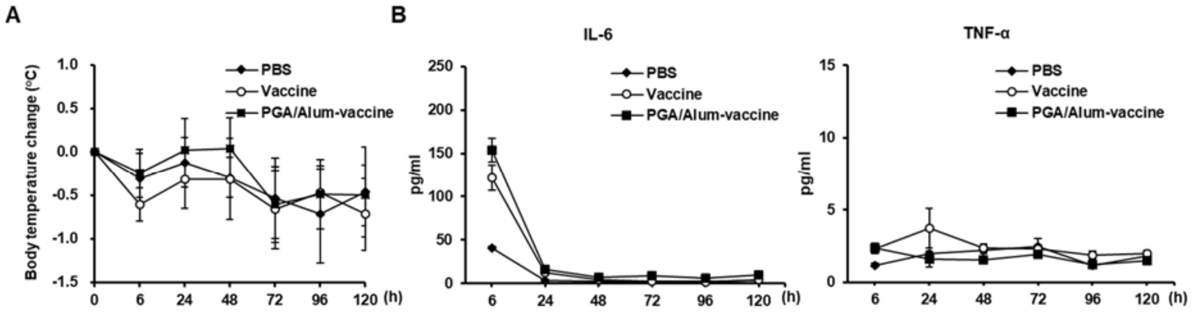

Supplementary Figure 2. Immunization of PGA/Alum-vaccine has no significant changes of systemic inflammation. C57BL/6 mice (*n* = 6 per group) were i.m immunized in tibialis anterior with PBS, 0.05 μg pH1N1 vaccine alone or combined with 800 μg PGA/Alum. At indicated time points, (A) body temperature was measured using a rectal probe and (B) levels of inflammatory cytokines (IL-6 and TNF-α) were determined in sera from the vaccinated mice using a Legendplex immunoassay kit. The data are presented as a mean ± SD.

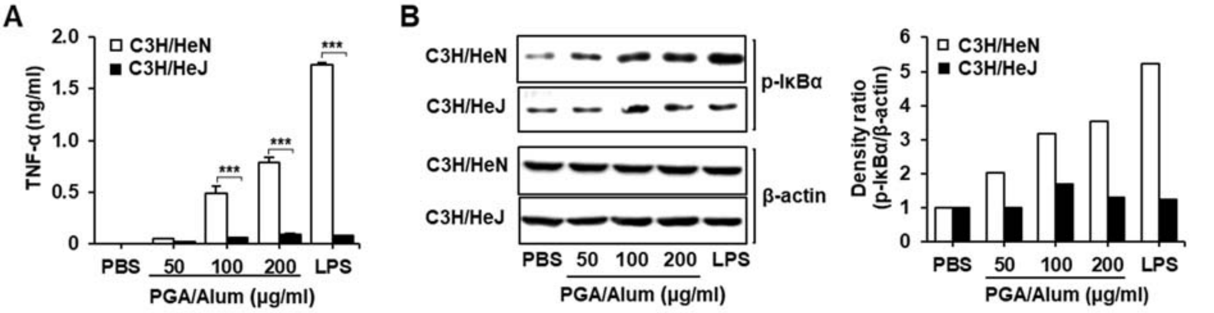

Supplementary Figure 3. PGA/Alum-induced DC activation is mediated through TLR4. Immature BMDCs were generated from C3H/HeN and C3H/HeJ mice. (A) The cells were stimulated with 50, 100, and 200 μg/ml PGA/Alum or 0.1 μg/ml LPS (as a positive control) for 24 h, and ELISA was used to measure TNF-α levels in culture supernatants. The data are presented as a mean ± SD. Statistically significant differences were analyzed by student's *t-test*; ***$P < 0.001$. (B) BMDCs were serum-starved for 3 h and stimulated with PGA/Alum at various concentration or 0.1 μg/ml LPS for 30 min. Phosphorylated IκBα and β-actin were determined by Immunoblotting, and band intensities were quantified by the ImageJ software. The data are representative of three independent experiments.

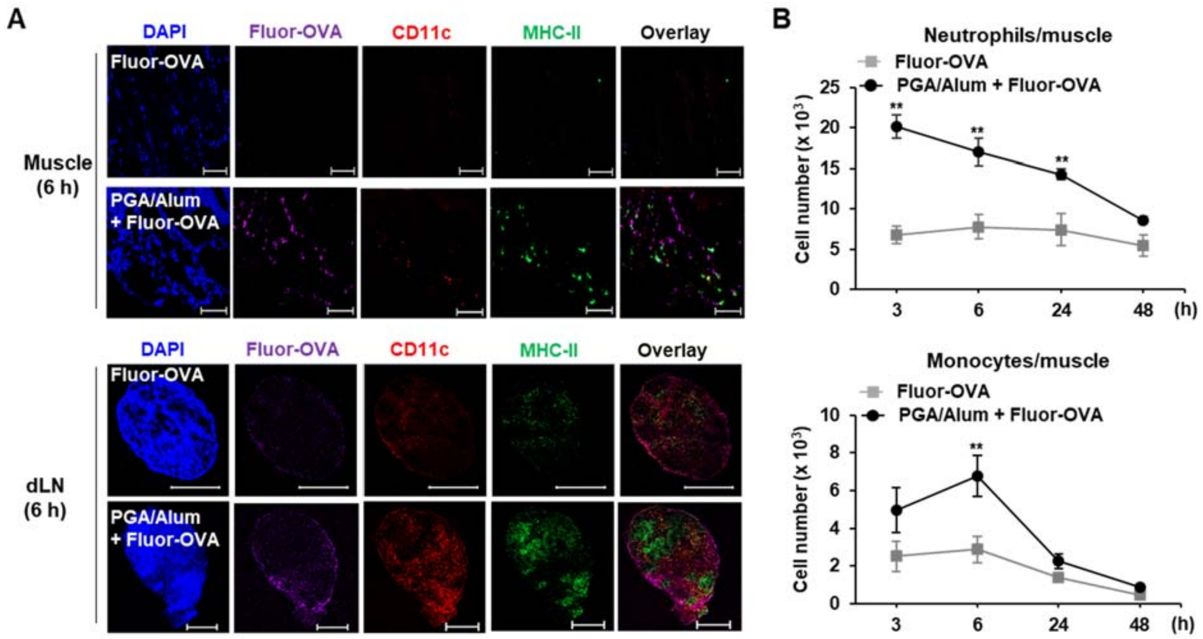

Supplementary Figure 4. PGA/Alum highly induces recruitment of DCs, neutrophils, and monocytes at the site of injection as well as accumulation of DCs at the dLN. C57BL/6 mice (n = 3 per group) were i.m immunized in tibialis anterior with 5 µg Fluor-OVA alone (purple) or combined with 800 µg PGA/Alum. (A) Immunofluorescent microscopic analysis was performed on frozen sections of injected muscles and dLNs obtained from mice at 6 h post-injection. Sections were stained with DAPI (blue) and antibodies against CD11c (red) and MHC-II (green) (scale bars, 50 µm for muscle and 500 µm for dLNs). (B) The number of neutrophils (gated as $SSC^{high}Ly6G^{high}$) and monocytes (gated as $CD11c^{-}CD11b^{+}Ly6C^{high}$) at the injected muscles was determined by flow cytometry. The data are presented as a mean ± SD and representative of three independent experiments. Statistically significant differences were analyzed by student's *t-test*; $^{**}P < 0.01$.

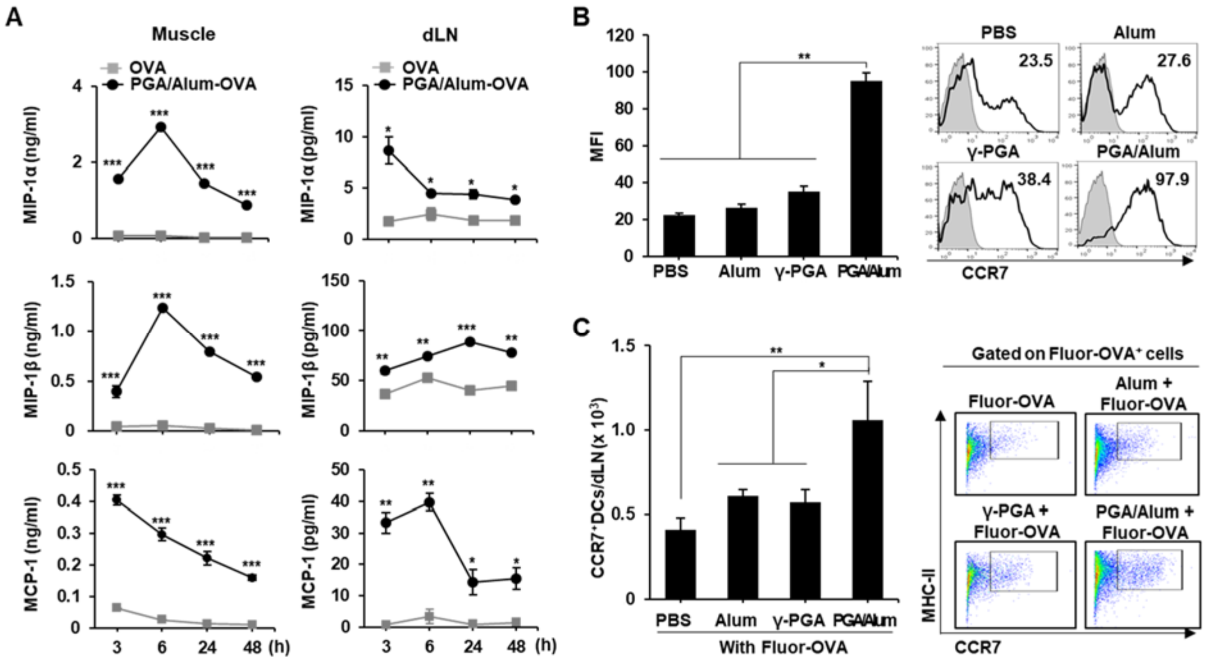

Supplementary Figure 5. PGA/Alum increases production of chemokines and expression of CCR7 on DCs. (A) C57BL/6 mice (*n* = 3 per group) were i.m immunized in tibialis anterior with 5 µg OVA alone or combined with 800 µg PGA/Alum. At 3, 6, 24, and 48 h post-immunization, levels of chemokines were determined in the tissue homogenates of the injected muscles and dLNs using a Legendplex immunoassay kit. (B) Immature BMDCs were treated with 100 µg/ml alum, 100 µg/ml γ-PGA, or 200 µg/ml PGA/Alum for 24 h. Expression of CCR7 on the BMDCs was determined by flow cytometry. The number in histogram indicates MFI values. (C) C57BL/6 mice (*n* = 3 per group) were i.m immunized in tibialis anterior with 5 µg Fluor-OVA alone or combined with 400 µg alum, 400 µg γ-PGA, or 800 µg PGA/Alum. Twenty-four hours post-immunization, expression of CCR7 on Fluor-OVA$^+$MHC-II$^+$ cells of the dLNs was determined by flow cytometry. The data are presented as a mean ± SD and representative of three independent experiments. Statistically significant differences were analyzed by ANOVA/Bonferroni; *$P < 0.05$, **$P < 0.01$, and ***$P < 0.001$.

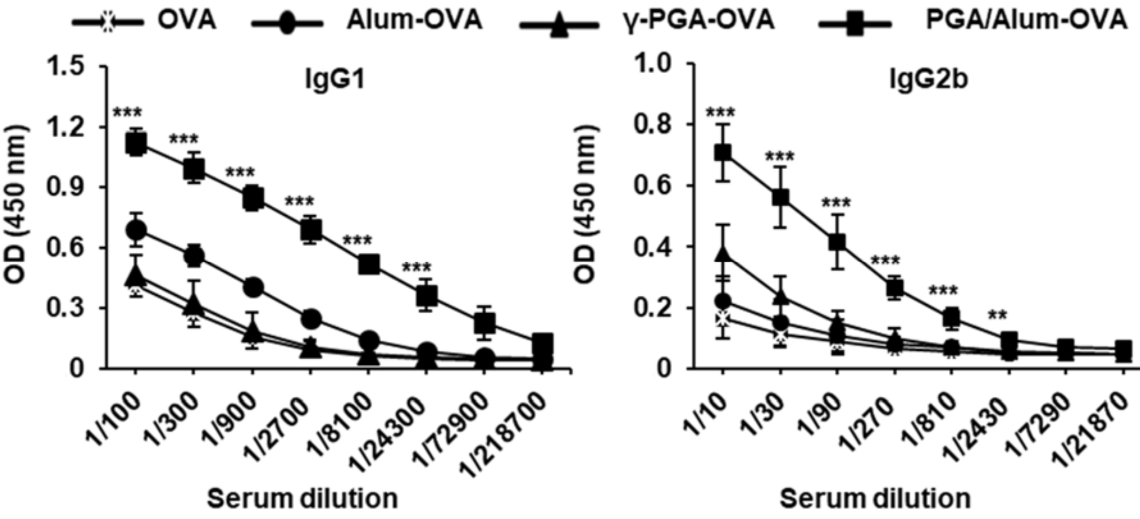

Supplementary Figure 6. PGA/Alum enhances levels of OVA-specific IgG1 and IgG2b antibodies. C57BL/6 mice were i.m immunized with 10 µg OVA protein alone or combined with either 400 µg alum, 400 µg γ-PGA, or 800 µg PGA/Alum on days 0, 14 and 28. Fourteen days after the last immunization, sera were obtained from the immunized mice and analyzed to determine levels of OVA-specific IgG1 and IgG2b antibodies by ELISA. Data are representative of three independent experiments with similar results. Statistically significant differences were identified by ANOVA/Bonferroni; $**P < 0.01$ and $***P < 0.001$.

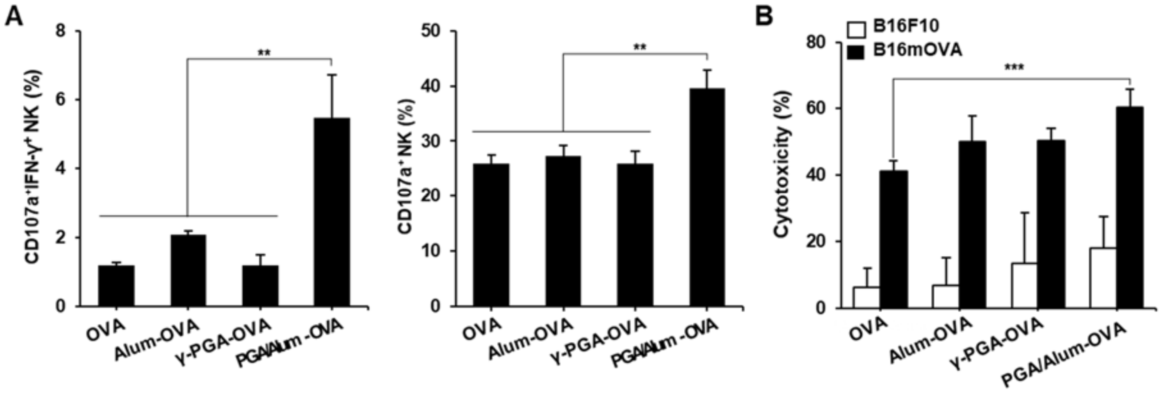

Supplementary Figure 7. Serum Abs of PGA/Alum-OVA-immunized mice effectively enhance NK cell activation facilitating ADCC activity. C57BL/6 mice (*n* = 5 per group) were i.m immunized with 10 µg OVA protein alone or combined with either 400 µg alum, 400 µg γ-PGA or 800 µg PGA/Alum 3 times at 2-week intervals. Fourteen days after the last immunization, sera were obtained from the mice and heat-inactivated at 56ºC for 1 h. (A) OVA-coated plates were incubated with the sera, washed, and then further incubated with naïve NK cells in the presence of PE-conjugated anti-CD107a mAb, monensin, and brefeldin A at 37ºC for 5 h. The cells were fixed, permeabilized, and stained with APC-conjugated anti-IFN-γ mAb. The stained cells were analyzed for expression of CD107a and IFN-γ by flow cytometry. (B) B16mOVA or B16F10 cells were incubated with heat-inactivated sera (56°C, 1 h) and naïve NK cells for 4 h, and the culture supernatants were subjected to LDH assays to detect ADCC-mediated cytolytic activity. The data are presented as a mean ± SD and representative of three independent experiments. Statistically significant differences were identified by ANOVA/Bonferroni; $^{**}P < 0.01$ and $^{***}P < 0.001$.

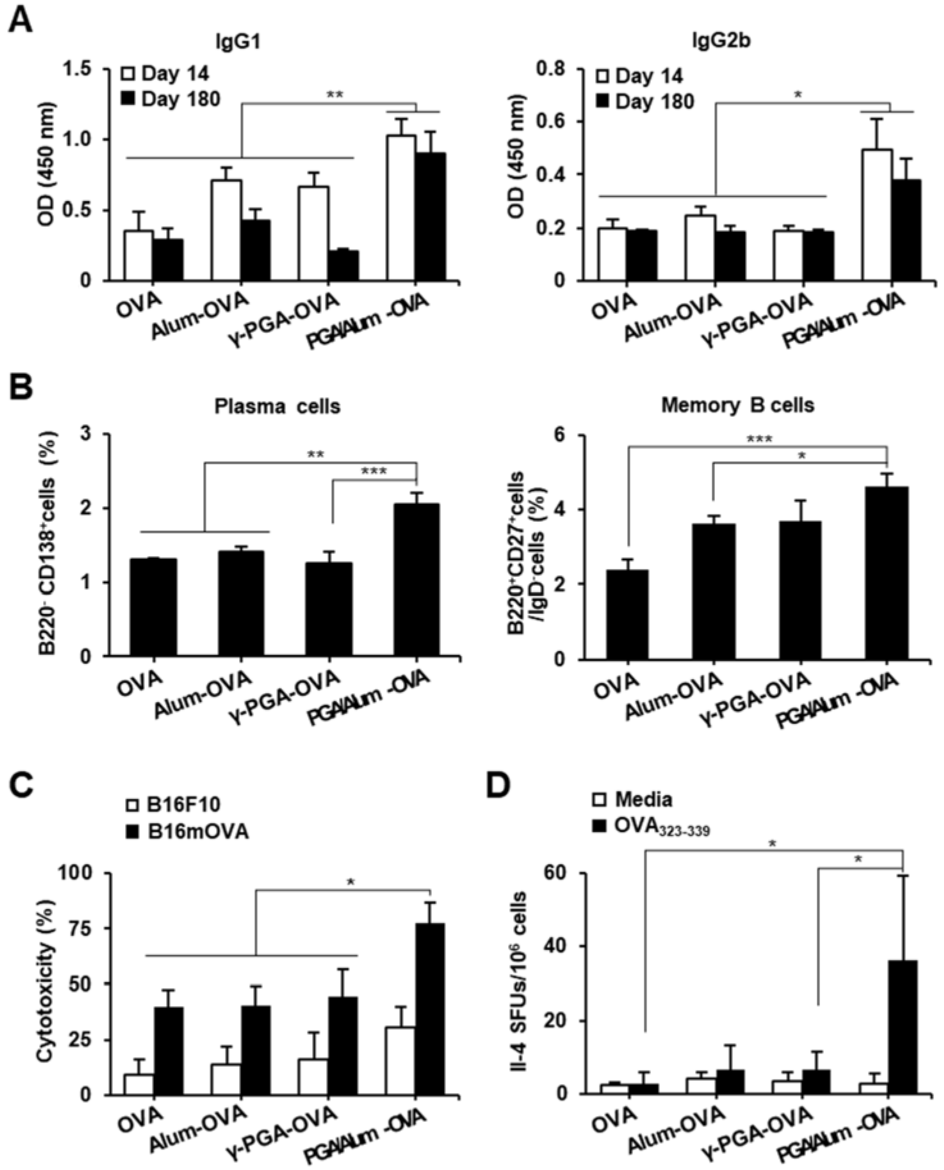

Supplementary Figure 8. PGA/Alum-OVA-immunized mice maintain long-lasting immune responses. C57BL/6 mice (*n* =5 per group) were i.m immunized 3 times at 2-weeks intervals with 10 µg OVA protein alone or with either 400 µg alum, 400µg γ-PGA, or 800 µg PGA/Alum. (A) Sera were isolated from the immunized mice on days 14 and 180 after the last immunization. Levels of OVA-specific IgG subclasses (IgG1 and IgG2b) were determined in the sera by ELISA. (B) Splenocytes from the immunized mice were obtained on day 180 post-immunization. Percentages of plasma cell populations (gated as $CD138^{+}B220^{-}$) and memory B cells (gated as $CD27^{+}B220^{+}IgD^{-}$) in the splenocytes were analyzed by flow cytometry. (C) B16mOVA cells were incubated with the sera (heat-inactivated, 56ºC, 1h) and naïve NK cells for 4 h at 37ºC. Culture supernatants were subjected to LDH assays for ADCC-mediated cytolytic activity. (D) Splenocytes were stimulated with 5 µg/ml $OVA_{323\text{-}339}$ peptides for 3 days, and the number of IL-4-secreting SFUs was measured by ELISPOT assay. The data are presented as a mean ± SD and representative of three independent experiments. Statistically significant differences were analyzed by ANOVA/Bonferroni; *$P < 0.05$, **$P < 0.01$, and ***$P < 0.001$.

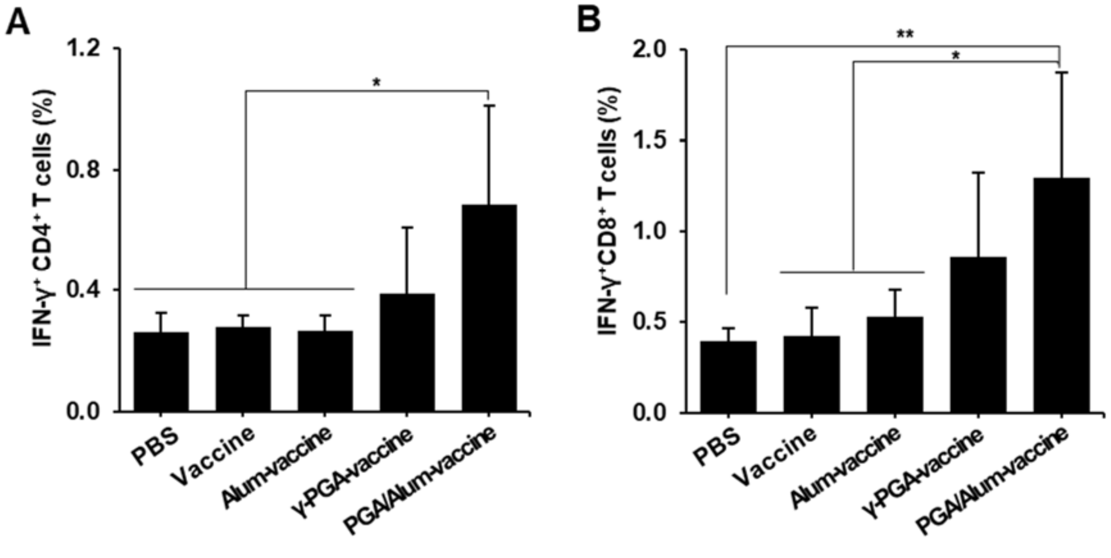

Supplementary Figure 9. PGA/Alum improves influenza virus antigen-specific T cell responses. C57BL/6 mice (*n* = 6 per group) were i.m. immunized with pandemic influenza vaccine alone or combined with 400 µg alum, 400 µg γ-PGA, or 800 µg PGA/Alum on days 0 and 14. Two weeks after the final administration, splenocytes were obtained from the immunized mice and stimulated with 500 $TCID_{50}$ UV-inactivated pH1N1 virus. Percentages of influenza virus antigen-specific IFN-γ^{+}CD4^{+} T cells (A) and IFN-γ^{+} CD8^{+} T cells (B) were analyzed by flow cytometry. Data are representative of three independent experiments. Statistically significant differences were analyzed by ANOVA/Bonferroni; *$P < 0.05$ and **$P < 0.01$.

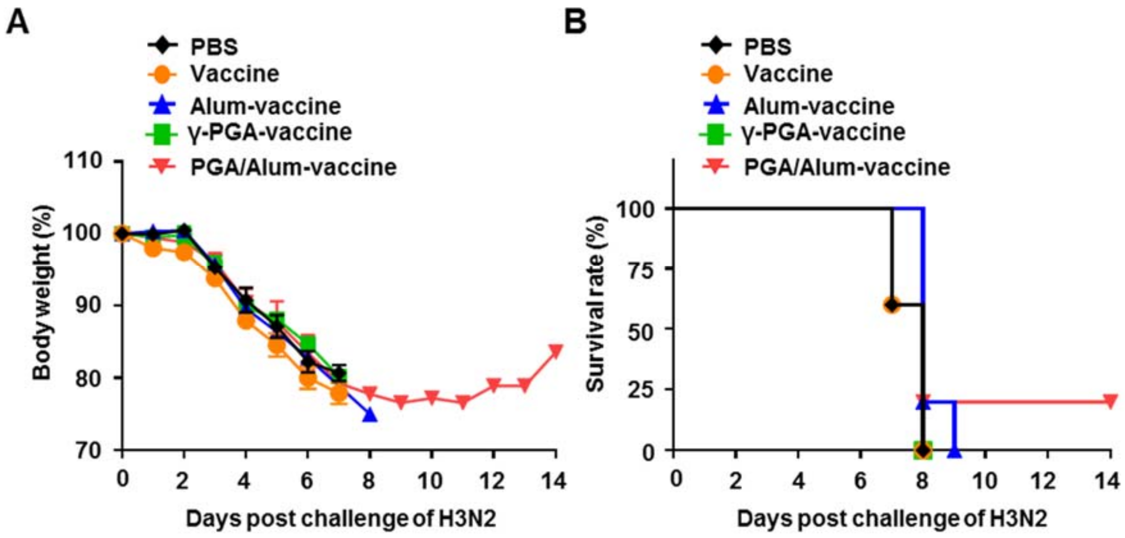

Supplementary Figure 10. Serum Abs from the PGA/Alum-pH1N1 vaccine group partially contribute to *in vivo* cross-protection against H3N2 virus. C57BL/6 mice (*n* = 5 per group) were twice immunized with 0.5 μg the pH1N1 split vaccine alone or combined with 400 μg alum, 400 μg γ-PGA, or 800 μg PGA/Alum at 2-week intervals. Two weeks after the final immunization, sera were obtained and heat-inactivated at 56°C for 1 h. The heat-inactivated sera were mixed with 2 LD_{50} H3N2 virus at room temperature for 30 min, and then Balb/c mice (*n* = 5 per group) were i.n. infected with the mixture. (A) Body weight and (B) survival rate were monitored for 14 days after the viral challenge.

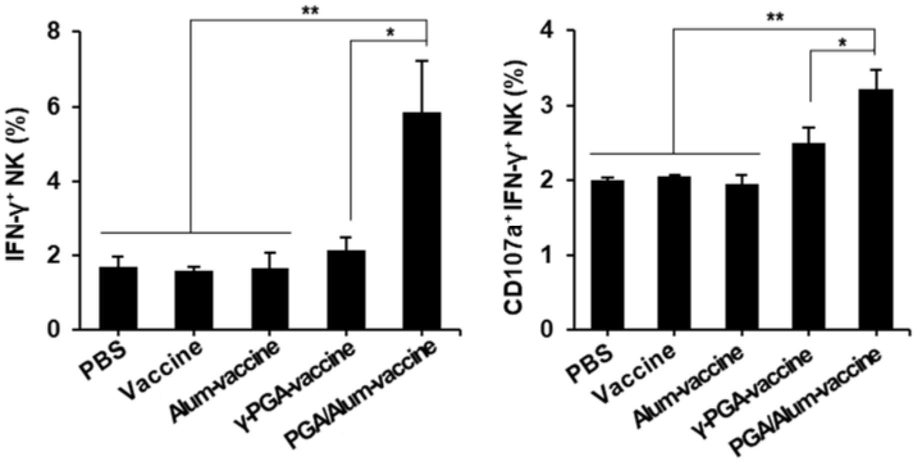

Supplementary Figure 11. Serum Abs from PGA/Alum-vaccine group drastically activate NK cells. C57BL/6 mice (n = 6 per group) were i.m. immunized with the pandemic H1N1 split vaccine antigen combined with 400 μg alum, 400 μg γ-PGA, or 800 μg PGA/Alum on days 0 and 14. Fourteen days after the last immunization, sera were obtained from the mice and heat-inactivated at 56°C for 1 h. HA stem region antigen-coated plates were incubated with the sera, washed, and then further incubated with naïve NK cells in the presence of PE-conjugated anti-CD107a mAb, monensin, and brefeldin A at 37°C for 5 h. The cells were fixed, permeabilized, and stained with APC-conjugated anti-IFN-γ mAb. The stained cells were analyzed for expression of CD107a and IFN-γ by flow cytometry. The data are presented as a mean ± SD and representative of three independent experiments. Statistically significant differences were identified by ANOVA/Bonferroni; $*P < 0.05$ and $**P < 0.01$.

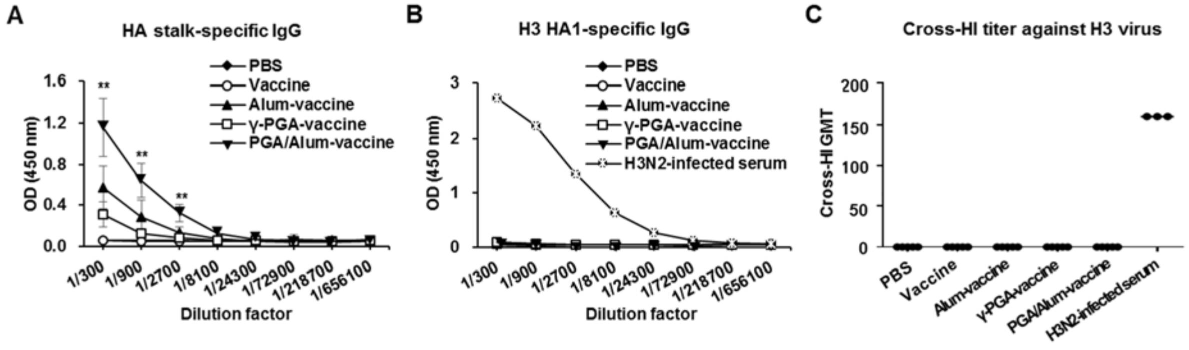

Supplementary Figure 12. PGA/Alum-vaccine-immunized mice increase the level of H1 HA stalk-specific Abs but not H3 HA1-specific Abs and cross-reactive neutralizing Abs. C57BL/6 mice (*n* = 5 per group) were twice immunized with 0.5 μg the pH1N1 split vaccine alone or combined with 400 μg alum, 400 μg γ-PGA, or 800 μg PGA/Alum at 2-week intervals. Two weeks after the final immunization, sera were obtained from the immunized mice. Levels of serum IgG specific to (A) HA stalk region of A/Puerto Rico/8/34 (H1N1) virus or (B) HA1 of A/HongKong/1/68 (H3N2) virus were determined by ELISA. (C) Serum HI titer against H3N2 virus were measured. Serum from the mice infected with 0.1 LD_{50} H3N2 virus was used as a positive control. The data are presented as a mean ± SD, and statistically significant differences were identified by ANOVA/Bonferroni; $^{**}P < 0.01$.
